# Taxonomic Classification of Bacterial 16S rRNA Genes Using Short Sequencing Reads: Evaluation of Effective Study Designs

**DOI:** 10.1371/journal.pone.0053608

**Published:** 2013-01-07

**Authors:** Orna Mizrahi-Man, Emily R. Davenport, Yoav Gilad

**Affiliations:** Department of Human Genetics, University of Chicago, Chicago, Illinois, United States of America; University of Illinois, United States of America

## Abstract

Massively parallel high throughput sequencing technologies allow us to interrogate the microbial composition of biological samples at unprecedented resolution. The typical approach is to perform high-throughout sequencing of 16S rRNA genes, which are then taxonomically classified based on similarity to known sequences in existing databases. Current technologies cause a predicament though, because although they enable deep coverage of samples, they are limited in the length of sequence they can produce. As a result, high-throughout studies of microbial communities often do not sequence the entire 16S rRNA gene. The challenge is to obtain reliable representation of bacterial communities through taxonomic classification of short 16S rRNA gene sequences. In this study we explored properties of different study designs and developed specific recommendations for effective use of short-read sequencing technologies for the purpose of interrogating bacterial communities, with a focus on classification using naïve Bayesian classifiers. To assess precision and coverage of each design, we used a collection of ∼8,500 manually curated 16S rRNA gene sequences from cultured bacteria and a set of over one million bacterial 16S rRNA gene sequences retrieved from environmental samples, respectively. We also tested different configurations of taxonomic classification approaches using short read sequencing data, and provide recommendations for optimal choice of the relevant parameters. We conclude that with a judicious selection of the sequenced region and the corresponding choice of a suitable training set for taxonomic classification, it is possible to explore bacterial communities at great depth using current technologies, with only a minimal loss of taxonomic resolution.

## Introduction

Originally proposed by Woese and Fox, the classification of ribosomal RNA genes has been the gold standard for molecular taxonomic research for decades [1], [2]. The 16S small ribosomal subunit gene (16S rRNA), in particular, has been widely used to study and characterize bacterial community compositions in a variety of ecological niches including host associated communities, such as the endogenous human microbiome [3]–[5], and host-free communities, such as soil and ocean environments [6], [7]. Several aspects of the 16S rRNA gene make it optimal as a marker for these types of studies. First, it is ubiquitous among prokaryotic life. Second, its size and high degree of functional conservation result in clock-like mutation rates throughout prokaryotic evolution [8]. Third, and most importantly, the 16S rRNA gene includes both conserved regions, which can be used for designing amplification primers across taxa, as well as nine hypervariable regions (V1-V9), which can be effectively used to distinguish between taxa [9].

Early bacterial community studies typically sequenced the entire 16S rRNA gene, but their ability to sample the full array of bacterial diversity was limited by depth of sequencing. With the advent of massively parallel sequencing technologies, which generally yield short reads, focus has shifted from sequencing the full 16S rRNA gene to sequencing shorter sub-regions of the gene at great depth [10]–[12]. Of the second-generation sequencing platforms, large-scale pyrosequencing (454) was the preferred choice initially, as it provides somewhat longer reads (up to 500 bp) compared to other platforms. However, recently, other platforms (such as the Illumina MiSEQ and HiSEQ) have become much more attractive for microbiome studies due to increase in sequencing read length (to ∼100 bp at the moment and up to ∼250 bp next year) combined with a much higher and cheaper output [10], [13]–[15]. Though the microbiome field is experiencing a shift in the choice of sequencing technology there has not been a systematic evaluation of the properties of alternative study designs that utilize these technologies.

Several studies have examined different aspects of short read study designs for 16S classification by analyzing the effects of variation in read length, region choice, and sampling depth [16]–[23]. However, results from these studies are often conflicting [16], [18]–[23] and a standard design for bacterial community studies has not yet emerged (e.g., different studies sequence different 16S rRNA gene regions). In addition, the rapid pace at which sequencing technologies are evolving requires frequent evaluation and modifications of the optimal study designs. A common framework that would facilitate the evaluation, comparison, and optimal parameterization of different experimental designs in terms of precision and coverage is not yet available.

There are three properties of bacterial community studies using short sequencing read technologies that principally determine the extent to which the study is effective. First, the specific sequencing strategy (e.g., read length, single or paired end); second, the choice of 16S rRNA gene regions to be sequenced; third, the choice of taxonomic classifier. Most studies of bacterial communities to date used 454 sequencing (relatively long reads, which can include more than one 16S rRNA gene hypervariable region, but relatively low depth). However, recently several studies have used deep, short-read, single-end Illumina sequencing, with few studies using paired-end sequencing but analyzing the data without taking into account the paired-end structure of the reads [10], [13], [14], [24]–[29]. With the rise in the use of Illumina short-read sequencing for bacterial community studies, it is important to now rigorously examine these three properties mentioned above in a systematic and comprehensive way to develop sequencing strategies appropriate for this platform.

The first property, sequencing strategy, has not been fully examined in the literature. Although several studies of 16S rRNA gene sequencing study design looked at various read lengths [11], [19], [21], many of these read lengths were chosen based on the capacities of 454 technology, and are not ideal for short, Illumina length reads. In addition, few studies have taken into account the ability for Illumina reads to be paired-end [11], [21], [29]. In a recent study, Werner et al [29] examined the merits of paired-end sequencing as compared to single-end sequencing, but rather than classifying their reads to a known taxonomy, the authors clustered their data into operational taxonomic units (OTUs) and calculated diversity indices, as well as built a phylogenetic tree from these OTUs. Finally, the limitations of insert size have not been considered in various studies. For example, Soergel, et al. [21] recently examined combinations of primers spanning the full region of the 16S rRNA gene, without taking into account that some of these products after amplification would be longer than the generally accepted 400–600 bp insert size recommendations for Illumina sequences.

Several bioinformatics studies have examined the second property, namely the choice of 16S rRNA gene region [11], [18]–[21], [23]. Most extensive among these was the recent study by Soergel et al [21], which examined thousands of primer and read length combinations, but unfortunately focused only on queries that had a close counterpart (at least 97% identity) in the reference database. As a result, there is no way of knowing how the experimental designs examined would perform on novel species, which are common in environmental surveys. This and other studies have come to differing conclusions on the most effective hypervariable region to target, with recommendations including one or more of the V2, V3, V4, V6 or V3/V4 regions. These differences in results are possibly due to many factors including specific primers examined, the environmental source of the reads, and classification method and parameters chosen during analysis. This lack of consensus is apparent in recent literature, with most current studies focusing on either hypervariable region V3, V4 or V6 [10], [28], [30]–[33], with no convergence on a single hypervariable region being chosen.

With regards to the third property, the choice of classifier, there are three varieties of algorithms to choose from. One category of tools, classifies 16S rRNA gene fragments based on sequence alignment based similarity. Notable in this category is the GAST [18] program, which was specifically designed with short gene fragments in mind. A second category of tools, including for example pplacer [34], attempts to find the correct location of the gene fragment within a phylogenetic tree of the reference sequences. The third category of tools uses word composition to assign taxonomy to a read. Algorithms in this category include nearest-neighbor searches as in SimRank [35] and SeqMatch [23] and naïve Bayesian classifiers, such as the ribosomal database project (RDP) classifier [23] and Mothur’s classify.seq function [36]. Liu et al [19] performed a comprehensive comparison among methods from all three of these categories (but did not include GAST [18] or pplacer [34], which had not published yet at the time) and recommended the RDP classifier [23] and SimRank [35] for the accuracy and stability of their results, with RDP classifier being the most efficient of the methods examined in terms of run time. Despite not being designed for short sequences, for a number of primers these classifiers showed excellent recovery and coverage even for 100 nt sequences [19]. Indeed, naïve Bayesian classifiers have been a popular choice for the analysis of high throughput sequencing reads (for example, [31], [37]–[39]). We therefore decided to focus on naïve Bayesian classifiers for our analyses.

As mentioned above, available naïve Bayesian classifiers were originally developed to classify full-length 16S rRNA gene sequences and have not yet been thoroughly evaluated and optimized for use with short sequencing reads. In particular, the default use of the RDP classifier utilizes a training set of full 16S rRNA gene sequences, which might be suboptimal for the purpose of classifying short reads. Indeed, it has been suggested that trimming the selected training set down from the full-length 16S rRNA gene to the size of the sequenced region improves the performance of the classifier [40].

There is also the issue of determining confidence score cutoffs for classification. Rather than reporting the probability that a prediction is correct, all current implementations of naïve Bayesian classifiers provide a confidence score for each level of the classification, with the intuitive interpretation of low scores being that a given sequence could belong to one of several taxa with almost equal probability. These confidence scores, however, cannot be directly translated to probabilities of error. The original recommendation of the RDP classifier developers, which is widely used in the literature, was to use a confidence score threshold of 80 regardless of the taxonomic level that is being classified, and this is the default threshold for this classifier [23]. Claesson et al. [16] compared the performance of this default threshold with a threshold of 50% in the classification of subsequences from three hypervariable regions, using a sample of near full-length sequences from the human gut. Subsequently the RDP developers adopted Claesson et al.’s [16] recommendation to use a threshold of 50 for the classification of 16S rRNA gene fragments of length 50–250 nt, regardless of the 16S rRNA gene region that is being sequenced (or the taxonomic rank) (http://rdp.cme.msu.edu/classifier/class_help.jsp#conf). Most studies published to date that used a naïve Bayesian classifier for taxonomic classification utilized 50% or 80% as a confidence threshold. Ultimately, we wish to understand confidence score thresholds in terms of estimates of false negative and positive rates, or false discovery rates. Confidence thresholds should be chosen that balance precision and coverage.

In this study, we synthesize ideas and analyses that cover the range of all three principle properties that determine effectiveness in study design, incorporating these various parameters into a unified, comprehensive study aimed at determining the most effective 16S rRNA gene based assays for short-read second generation sequencers. We present a bioinformatic analysis, using a naïve Bayesian classifier, of various study designs for short read bacterial community experiments, exploring different sequencing strategies, 16S rRNA gene regions, and training sets, and examining the relationship between confidence scores, false discovery rates, and overall coverage. We offer recommendations for an effective bacterial community study design given the capabilities of current technologies, as well as propose a framework that allows one to optimize the parameters of the study design when new technologies emerge.

## Results and Discussion

### A Framework for the Comparison of Classification Performance

Our goal was to compare the performance of bacterial community study designs using different regions of the 16S rRNA gene to classify sequences into known bacterial taxa. Previous studies evaluating alternative experimental designs did so using one [11], [18], [19] or very few parameter settings [16], [21]. Optimal classification performance balances precision and coverage. Therefore, to obtain a complete view when comparing the effectiveness of different experimental designs we wished to evaluate their performance while varying the classifier parameters. To this end we devised a framework in which classification performance was evaluated for both precision and coverage. In the current study we used this framework to evaluate the classification by the naïve Bayesian classifier of sequencing configurations typical of the current capabilities of the Illumina platform. However, the same evaluation framework can be generalized and applied to any classifier and experimental configuration.

The first consideration in the framework we propose is a stringent method to evaluate the precision of the classifier being tested. A typical approach is the leave-one-out cross-validation method, which requires just one set of sequences. In each iteration all sequences but one are used for training, and the sequence left out is being classified [19], [23]. However, the training set is a parameter of the classifier, for which one may wish to evaluate several options. For a fair comparison in our study, we need to settle on one “test set” with known taxonomy that can be used across all tests. We selected the ∼8,500 bacterial sequences and their corresponding taxonomic annotation from “The All-Species Living Tree” project (LTP; [41], [42]) as a test set, considering each time only the subsequences relevant for the experimental design in question. Then, to evaluate precision we used a leave-k-out approach, whereby, in each iteration, we removed from the training set all sequences of the species to be classified (see Methods for more details). This is a rather conservative approach, as previous studies excluded from training only the particular tested sequence [19], [23]). In addition, We also included all sequences in the evaluation, regardless of their distance from the closest match in the training database (instead of following the common approach of retaining only sequences with at least 97% identity with the closest match [21]). In turn, to evaluate and compare coverage across the different training sets, we used a set of more than a million environmental (uncultured) bacterial sequences (available through the RDP database [43], [44]), focusing on the subsequences relevant to the experimental design (see Methods for more details).

The second major aspect of our framework was to assess the precision of the classifier in terms of false prediction rates of classification of our sequences. The naïve Bayesian classifier accompanies each prediction with a bootstrap confidence value. However, the same bootstrap value obtained by the naïve Bayesian classifier may result in different precision and coverage for different taxonomic ranks, as well as for different regions [16], [19]. Thus, to obtain a complete view of the relationship between precision and coverage we explored in this study the full range of bootstrap confidence values. In the analyses that follow, the distribution of confidence values obtained in the leave-k-out tests was skewed towards high confidence values (Figure S1), probably because most test sequences are associated with densely sampled regions of bacterial phylogeny. In order to draw conclusions that would be applicable to any set of sequences, irrespective of the distribution of confidence values, we used a pre-defined set of precision values and determined conservative thresholds that would ensure these precisions at a minimum (see Methods). We then used these thresholds to compute coverage in environmental sequences.

### Diverse Training Sets Afford Better Performance

The basis for taxonomic classification of unknown 16S rRNA gene sequences is a training set of sequences with known taxonomy. We tested out several different training sets in order to select the set with best performance for short reads. We reasoned that the optimal training set would be current and accurate in terms of underlying taxonomy, as well as diverse, containing sequences representing all bacteria cultured to date. We compared the performance of the RDP training set v. 6 (‘RDP TS6’), which is the built-in training set for RDP classifier [23] and thus the most commonly used training set when classifying 16S rRNA genes using a naïve Bayesian classifier, to that of three alternative sets (Table 1). ‘RDP TS6’ is comprised of ∼8,500 mostly bacterial, type strain sequences, and therefore includes only a fraction of the diversity currently available in 16S rRNA gene databases. The bacterial portion of the taxonomic hierarchy underlying this training set was last updated in 2008 [44].

**Table 1:** Training sets used for the naïve Bayesian classification of bacterial 16S rRNA gene sequences.

Abbreviation	Description	Sequence Database^a^	Underlying Taxonomy
RDP TS6	RDP classifier training set v.6 (default forv. 2.3 of the RDP classifier)	8,127 bacterial and 295 archaeal sequences	Based on “The Taxonomic Outline of Bacteria and Archaea” (TOBA) 7.7 [52]
LTP	Bacterial subset of “The Living TreeProject” v. 106	8,494 bacterial sequences	“List of Prokaryotic names with Standing in Nomenclature (LPSN; http://www.bacterio.cict.fr/)
unfiltered RDP	All bacterial isolates in RDP database	31,334 non-redundant bacterial sequences^b^	Based on “The Taxonomic Outline of Bacteria and Archaea” (TOBA) 7.7 [52]
filtered NCBI	All bacterial isolates in RDP database,filtered for annotation quality	21,240 non-redundant bacterial sequences^b^	NCBI taxonomy [48]

a Except for the ‘RDP TS6’ training set, which always trains on the full sequence, numbers are only for the testing of 100 nt single-reads from the V4 region. For the three other training sets, which train only on the region to be classified, the number of sequences reflects both the number of sequences covering this region (all three training sets) and its degree of redundancy (‘unfiltered RDP’ and ‘filtered NCBI’).

b The numbers are for the ‘original non-redundant training set’ (see Methods section ‘Leave k out classification testing’); numbers for each leave-k-out iteration may vary slightly.

The first alternative, the living tree project (LTP) training set, was comprised of the ∼8,500 bacterial sequences used as a test set for precision, and emphasized both quality and currency, with diversity comparable to that of the ‘RDP TS6’. Second, the ‘unfiltered RDP’ training set was comprised of all bacterial isolate sequences available in the RDP database [43], [44]. This training set thus had the same taxonomic hierarchy as the ‘RDP TS6’ and is highly diverse. Third, the ‘filtered NCBI’ training set was comprised of the same sequences as the ‘unfiltered RDP’ training set, but was filtered for quality and currency of annotation (see Methods) and used the NCBI taxonomic annotation. This set is potentially the most current and accurate of all three, yet only slightly less diverse than the ‘unfiltered RDP’ training set. Finally, in contrast to ‘RDP TS6’, which is comprised of full-length sequences, all three alternative training sets were comprised of only short, partial 16S rRNA gene sequences (in our case, the sequences corresponding to the region to be classified).

**Figure 1:**
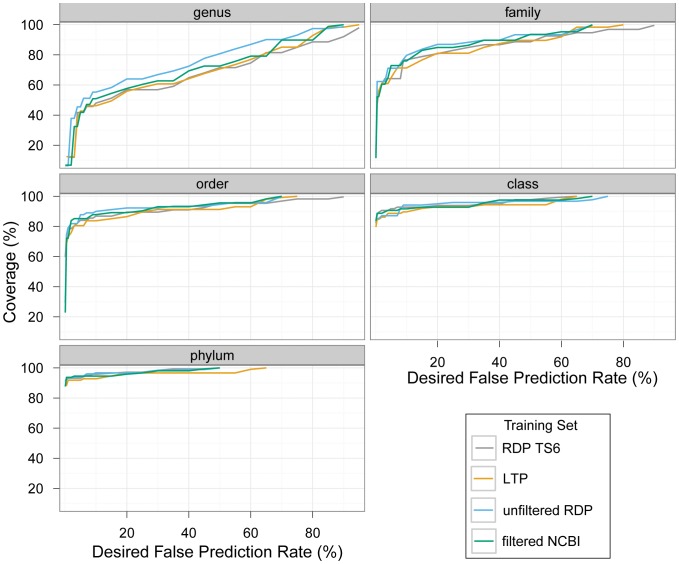
Performance of different training sets in the classification of 100 nt reads from the V4 amplicon. Each panel compares the performance of the training sets (described in Table 1) for a different rank. We used the results of leave-k-out tests classifying the LTP sequences to determine confidence score thresholds for a set of desired false prediction rate (FPR) values (x axis), so that the FPR would be at most the desired value. We then used these thresholds to calculate the classification coverage of sequences from environmental (uncultured) bacteria that corresponds to the desired FPR (y axis).

We used our evaluation framework to compare the performance of these four training sets in the classification of subsequences corresponding to the first 100 nt of the V4 amplicon. We found that training using the ‘unfiltered RDP’ set resulted in the highest genus-level prediction rate throughout the entire range of precision values (Figure 1). In contrast, the least diverse training sets (‘LTP’ and ‘RDP TS6’) had the worst performance, both performing almost equally. The order of performance across training sets was maintained for the most part at lower-resolution ranks (phylum, class, etc.), with the absolute differences in performance across training sets becoming smaller as taxonomic resolution decreases (as might be expected; Figure 1).

Werner, et al. [40] observed improved performance of the training set when trimming reference sequences to the region tested, compared to using the full 16S rRNA gene sequence as reference. However, our comparison of the performance of training sets on the V4 region does not support this notion. One of the main differences between the ‘LTP’ training set and the ‘RDP TS6’ was that the sequences in the former were trimmed to the region to be classified, whereas the latter contained full-length sequences. Nevertheless, these two training sets displayed similar performance. As an additional assessment of the effect of trimming of training set sequences, we compared the performance of training sets composed of sequences trimmed to the exact region used for classification (i.e. the region that would be sequenced) or to the complete amplicon (∼250 nt; see Methods). The two trimming regimes yielded practically identical results (Figures S2, S3 and S4). This observation indicates that, at least for the particular region we examined (V4), the composition of relatively short sequence stretches in the vicinity of the region used for classification is not a confounding factor. Because we had only compared trimming regimes in one region and in other regions using the full sequence or complete amplicon might still contribute noise, in downstream analyses we chose to trim the training sequences down to the region used for classification.

To further examine the performance of these four training sets and confirm our choice we sequenced, using Illumina technology, 102 bp single-end reads of the V4 region from two human fecal samples. We classified the reads using each of the four training sets, setting for each training set and rank a confidence threshold that would ensure at most 5% false predictions (Table 2; see Methods). While coverage differed significantly between the two samples, the ‘unfiltered RDP’ obtained the highest or second-highest coverage for all ranks but class (nine and three cases, respectively). Differences in performance among the training sets were quite dramatic at the higher-resolution ranks, especially at the genus level where training sets differed in performance by as much as 26% in the classification of sample B reads.

**Table 2:** Comparison of classification coverage of V4 reads from fecal samples among different training sets.

	Genus			Family			Order			Class			Phylum		
Training set	DB	A	B	DB	A	B	DB	A	B	DB	A	B	DB	A	B
RDP TS6	41.7	46.2	29.6	64.2	73.8	59.0	84.1	95.8	79.3	91.0 a	99.1 a	84.6 a	93.1	99.7	86.3
LTP	42.6	44.3	30.8	64.8	66.1	54.5	80.5	94.5	75.6	88.7	98.7	90.9	91.8	99.8	93.4
Unfiltered RDP	45.5	55.5	55.6	71.2	83.6	72.1	84.8 a	96.5 a	82.3 a	87.1	96.7	82.3	93.9	99.9	94.7
Filtered NCBI	41.9	46.8	38.8	72.9	78.2	61.4	85.2	96.4	79.2	90.7	97.5	85.6	94.5	99.9	94.7

We used each of the four training sets to classify single 100 bp reads excised from environmental (uncultured) bacteria 16S rRNA gene sequence from the RDP database (DB), as well as single100 bp reads from the same region sequenced from two fecal samples: A (6,298,382 sequences) and B (3,452,321 sequences). We then computed coverage for each of the ranks: phylum, class, order, family and genus, using per-rank confidence score thresholds that would ensure an FPR of at most 5%. The highest coverage in each column is underlined.

a The confidence score threshold for these cases was lower than that of a higher level/s, and a sequence could thus be classified at the current level but not at the higher taxonomic levels. We found that the classification of such sequences is associated with a high error rate and our recommendation is to exclude them. We have therefore adjusted coverage accordingly.

Thus, in line with the results of Werner, et al. [40], we found that the defining feature of a good training set is its diversity. Even a current, quality-filtered and relatively diverse training set (‘filtered NCBI’) performed poorly compared with the most diverse training set, which is less current and unfiltered for quality (‘unfiltered RDP’). Similarly, the ‘LTP’ training set and the ‘RDP TS6’, which contained similar numbers of sequences, performed equivalently, despite the fact that the taxonomy of the former is more current. This is perhaps because the sequence data available for novel taxa are too sparse. As more near-full-length sequences accumulate for the novel taxa it may become important to use a current taxonomy.

Given these observations, for subsequent exploration of different parameters of bacterial community study designs, we used the ‘unfiltered RDP’ training set, restricting the training in each case to the short 16S rRNA gene region that would be sequenced.

### False Prediction Rates are Dependent on Phylogenetic Resolution, Read Length and Gene Region

**Figure 2:**
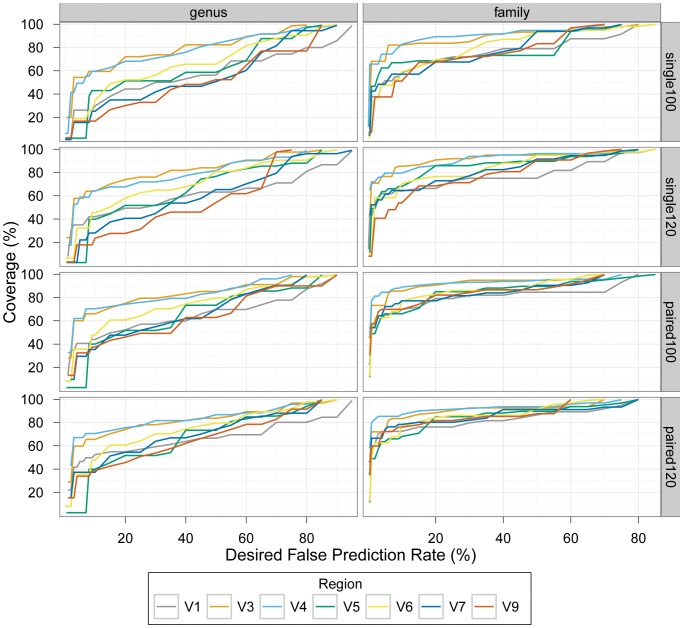
Classification performance of different experimental designs. Each panel compares performance of different regions for a different combination of rank (genus or family) and sequencing strategy (100/120 nt single/paired-end reads). We used the results of leave-k-out tests classifying the LTP sequences to determine confidence score thresholds for a set of desired false prediction rate (FPR) values (x axis), so that the FPR would be at most the desired value (Tables S4, S5, S6, S7, S8, S9, and S10). We then used these thresholds to calculate the classification coverage of sequences from environmental (uncultured) bacteria that corresponds to the desired FPR (y axis). Figure S5 compares the performance of different regions across the same sequencing configurations for the ranks order, class, and phylum.

Having selected the source for a training set, we turned to evaluate different short-sequencing reads bacterial community study designs. We considered seven different possible amplicon designs, derived from the 16S rRNA gene, each containing at least one hypervariable region (Table S1). We further examined four sequencing strategies of the different amplicons (appropriate for current technology): 100 nt single reads, 120 nt single reads, 100 nt paired-end reads, and 120 nt paired-end reads (Figures 2 and S5). To test the performance of different study designs we used the same approach we introduced above, evaluating and comparing precision and coverage for each configuration.

Like others [16], [19], [23], we found that the error rate associated with a confidence threshold is dependent on several factors, including the resolution of the prediction (e.g., phylum vs. genus), the length of sequence used for classification, and the region of the 16S rRNA gene from which the sequence was extracted. Consequently, the use of one overall ‘confidence’ threshold for classification (as commonly done) often results in suboptimal and unequal performance across regions and taxonomic ranks. For example, at a fixed confidence level threshold of 50 (recommended for short sequences by Claesson et al. [16]), the coverage for 100 nt single-reads varied from 52% (V9 when classifying genus) to 97% (V3 and V4 when classifying phylum), while the precision ranges from 45% (V5 and V7 when classifying genus) to ∼100% (V9 when classifying phylum). These observations indicate that an optimization of the confidence level threshold is required for each study design and taxonomic resolution. Evaluations of performance across study designs, in particular, may not be useful if a fixed confidence level is used as the basis for comparison rather than a similar level of coverage and/or precision. Therefore, confidence threshold cutoffs should be chosen that take these factors into account. For example, for classifying 100 nt single reads from the V3 region at the genus level (with error rate up to 5%) we recommend using a confidence threshold of 95% (Table 3). Using confidence thresholds 80% or 50% would result in error rates as high as 20% or 40%, respectively, depending on the distribution of confidence scores observed. On the other hand, for the same region and sequencing configuration the appropriate confidence threshold for achieving the same error rate when classifying phylum is 60%, resulting in 96% coverage. In this case, using the previously recommended confidence threshold of 80% would result in coverage of only 93%. Thus, our results underscore the need for a rank-specific and region-specific selection of the confidence threshold, depending on the desired false prediction rate (FPR). We provide a tabulation of recommended thresholds and coverage for a representative group of desired FPRs for all ranks, regions and configurations examined (Tables S4, S5, S6, S7, S8, S9 and S10).

**Table 3:** Recommended experimental designs.

		Primer pair		Genus		Family		Order		Class		Phylum	
Sequencingconfiguration	Region	Forward	Reverse	CT^b^	Coverage^c^	CT^b^	Coverage^c^	CT^b^	Coverage^c^	CT^b^	Coverage^c^	CT^b^	Coverage^c^
100 nt single	V3	F343^a^	R534	95	54	95	68	60	92	50	95 f	60	96
	V4	F515	R806 a	90	49	80	74	60	88 f	80	90	65	95
120 nt single	V3	F343^a^	R534	95	58	90	77	70	91	55	95	50^d^	97
	V4	F515	R806^a^	90	59	80	79	60	91	50	95 f	55	96
100 nt paired	V3^e^	F343	R534	95	60	95	73	75	92	55 d	96 f	60 d	96
	V4	F515	R806	95	62	85	84	70	93	70	95	45^d^	98
120 nt paired	V4	F515	R806	95	67	85	85	80	92	80	94	45^d^	98

a Primer would be used only for amplification, not for sequencing.

b The lowest confidence value threshold (CT) that is consistent with an FPR of 5% (see methods).

c Coverage (in percentage units) observed for the confidence threshold in environmental sequences.

d Median number of predictions in the interval [CT.CT+4] was smaller than 10.

e Results for 100 nt and 120 nt paired end configurations were practically identical for this region, as we encountered few V3 amplicons that were longer than 100 nt (all in the environmental sequences).

f CT for these cases is lower than that of a higher level/s, and a sequence can thus be classified at the current level but not at the higher taxonomic levels. We find that the classification of such sequences is associated with a high error rate and our recommendation is to exclude them, and have adjusted coverage accordingly.

### Superior Performance of Study Configurations Encompassing the V3 or V4 Regions

We next turned to compare amongst the different experimental designs, based on their evaluations in our framework (Figures 2, S2). As expected, we found that performance varied widely across different regions, sequencing strategies, and ranks. For example, at a 100 nt single read strategy the error rates associated with a confidence threshold of 0 (which maximizes coverage) across 16S rRNA gene regions were 80–95% for genus, 70–85% for family, 60–80% for order, 40–75% for class, and 35–70% for phylum. If we fix the classification precision level we can compare coverage across different study designs. At a precision of 95% (false prediction rate (FPR) of 5%), the genus-level coverage for 100 nt single-end reads varied from 2% (V5) to 54% (V3). A study design using V6, which is commonly used in short read sequencing, results in less than 20% coverage for genus classification when the required precision is 95% (Figure 2). In turn, if we maximize coverage across study designs we can compare precision levels; the maximal genus-level FPR for 100 nt single-end reads varies from 75% (V3 and V9) to 95% (V1 and V6). Our observations clearly show that the optimum balance between precision and coverage across 16S rRNA gene regions and taxonomic ranks is achieved at different confidence thresholds (Table 3).

With the exception of the short V5 amplicon (108 nt in the *E. coli* gene), performance substantially improved when we turned to consider study designs with longer sequencing strategies. Excluding V5, genus-level coverage obtained for 120 nt paired-end reads (at FPR = 5%) ranged from 34% (V9) to 67% (V4). Differences in performance between designs using different regions diminished with decreasing taxonomic resolution. For example, with 100 nt paired-end reads, the coverage at FPR ≤5% was 3%–62%, 63%–84%, 75%–93%, 88%–96%, and 93%–98%, for ranks genus, family, order, class, and phylum, respectively. Put together, our analysis suggests that the most effective study design utilizes paired end sequencing of V3 or V4 amplicons (Figure 2). In addition, consistent with previous reports [16], [19], we found that the V6 region, the region of focus in most bacterial community studies using short-read sequencing to date [5], [6], [28], [30], [39], does not perform well when a naïve Bayesian classifier approach is used, especially for classifying high-resolution taxonomic ranks. In contrast, using the GAST classifier, Huse, et al. [18] found both the V3 and V6 regions to have similar performance (no other regions were compared).

**Figure 3:**
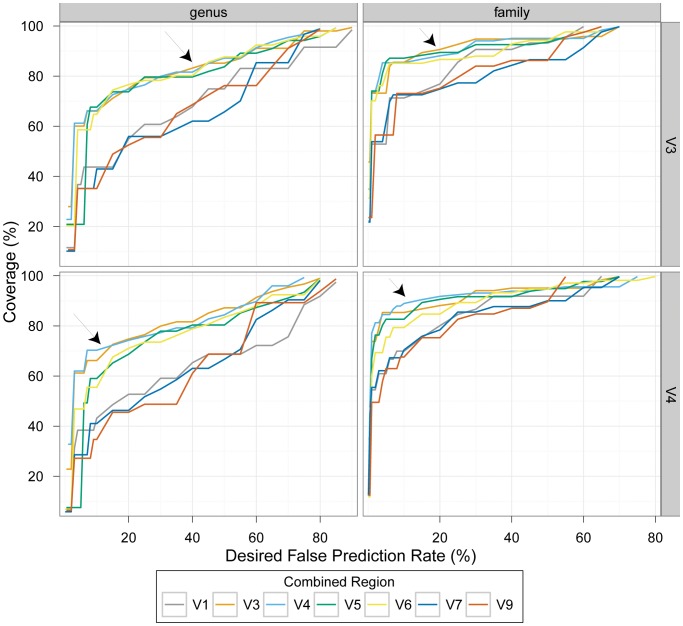
Classification performance of combined 100 nt single-read predictions, as compared to the best performing paired-end configurations. We combined predictions made for different 100 nt fragments of the same sequence, by selecting the prediction with the highest confidence score at the genus level (or the lowest common level available). We evaluated the performance, at ranks genus and family (left and right panels, respectively), of combinations of fragments from the V3 and V4 regions (top and bottom panels, respectively) with fragments from each of the other regions examined, and compared it to the performance of the V3 and V4 100 nt paired-end configurations (pointed to by arrows). We used the results of leave-k-out tests classifying the LTP sequences to determine confidence score thresholds for a set of desired false prediction rate (FPR) values (x axis), so that the FPR would be at most the desired value. We then used these thresholds to calculate the classification coverage of sequences from environmental (uncultured) bacteria that corresponds to the desired FPR (y axis). Figure S6 compares the performance of the combinations for the ranks order, class, and phylum.

Finally, to extend our analysis to study designs that might be practical in the near future, we asked if we could improve performance, whilst sequencing the same number of bases, by combining single-end reads across amplicons of different hypervariable regions instead of using a paired-end sequencing configuration. To this end we combined the predictions obtained using 100 nt single reads from each of the best performing regions (V4 and V3) with those obtained using each of the other hypervariable regions. We selected for each test sequence the prediction with highest confidence score at the genus level, with the assumption that it was known that the two predictions result from fragments of the same molecule (see Methods for more details). This is an idealized scenario, not attainable using current sequencing technologies. Regardless, combined predictions across multiple regions did not result in an overall improvement compared to study designs using 100 nt paired-end configurations of V3 or V4 (Figures 3 and S6).

Our analysis suggests that studies will do equally well focusing on either V3 or V4, irrespective of whether they choose a single or paired-end sequencing strategy. Yet, it should be noted that Youssef, et al. [45] reported that while species-richness estimates based on V4 are comparable to those from nearly full-length 16S rRNA gene sequences, analyses of V3 sequences tended to underestimate species richness. Regardless, the most effective experimental designs based on our analysis utilize the V3 or V4 paired-end sequence configuration. These designs are more effective even compared to the paired-end sequence configuration that overlaps both V7 and V9, and are comparable or more effective than all the configurations that combined single-read predictions of the V3/V4 regions with those from each of the other 16S rRNA gene regions (see Methods and Figures 3 and S6). Partly, this observation may be explained by the high coverage of V3 and V4 in the training set. Because not all 16S rRNA gene reference sequences in the training set are of full length, some regions of the molecule represent higher diversity than others. For example, V9 is the region with the least coverage in our database of reference sequences and correspondingly, study designs using the V9 regions performed poorly.

In our *in silico* study we made the unrealistic assumption that the sequences to be classified contained no errors and thus our results should be considered best-case scenarios. A good filtration protocol applied to the reads prior to classification, as well as filtering results based on their frequency of appearance, may substantially reduce the effect of sequencing errors in real data [10], [25], [27]. Yet, it is important to note that filtering paired-end reads is likely to result in the removal of a higher number of pairs compared to filtering single end reads. For that reason, the advantage we found for the paired-end sequencing strategy should be considered best-case scenario (see Werner et al., [29] for a detailed analysis).

In addition, it should be remembered that we assumed an ideal experimental system in which all primers are universal to bacteria with no bias. Before any of the primers examined in our study are utilized in an experimental setting care should be taken that they are appropriate for the probed environment. That said, the primer pair used here for the amplification of the V4 region (F515+R806) has been optimized for broad coverage [46], such that it is nearly universal.

### Conclusion

Based on our analysis, we recommend a focus on hypervariable regions V3 or V4 for interrogating bacterial communities with either single-read or paired-end strategies using 100/120 nt reads (Table 3). If a naïve Bayesian classifier is used, we recommend that appropriate confidence thresholds be selected for the classification of different taxonomic ranks such that the precision and coverage of the classified sequences are optimized. Our recommendations are relevant to study designs that are applicable to currently available sequencing technologies. As new technologies become available, our analysis platform can be used to explore and optimize different study design parameters.

## Materials and Methods

### Ethics Statement

The use of samples from human subjects in this study was approved by the University of Chicago IRB (protocol #10-416-B). All samples were collected with written informed consent.

### Training Sets

The ‘**RDP TS6**’ training set consists of the sequences and taxonomy of the training set v. 6 used by the RDP classifier [23] v. 2.3, which we downloaded on August 31, 2011 from the mothur [36] site (http://www.mothur.org/wiki/RDP_reference_files). We inferred the ranks of the various taxonomic path components based on the RDP classifier [23] version 2.3 hierarchy, which we downloaded from http://sourceforge.net/projects/rdp-classifier. To determine the species classification corresponding to the sequences, we used the annotation in the headers of the sequences of the SSURef subset of Release 108 of the SILVA database [47], downloaded from http://www.arb-silva.de/no_cache/download/archive/release_108/Exports/on September 1, 2011. For the few training sequences missing from the SSURef dataset, we obtained the species name manually from the NCBI website [48].

The ‘**unfiltered RDP**’ and ‘**filtered NCBI**’ training sets are based on bacterial isolate sequences from the RDP database [43], [44]. We used the RDP browser (http://rdp.cme.msu.edu/hierarchy/hb_intro.jsp) to download, on February 25, 2011, sequence alignment and annotation data for 250,706 high quality 16S rRNA sequences amplified from bacterial isolates, utilizing the nomenclatural taxonomy.

We obtained the taxonomic annotation of the ‘**unfiltered RDP**’ training set from the RDP database [43], [44] records, relying on the RDP classifier hierarchy for inference of the rank of taxonomic path components. In some cases, we found that the same name is given to different levels of the hierarchy (e.g. “Actinobacteria” is both the name of a phylum and a class). To obtain the rank in these cases we relied on the parent-child relationships specified by the hierarchy and the order of names in the taxonomic path. We used the Bio::LITE::Taxonomy::NCBI module version 0.06 to extract the species name corresponding to the NCBI taxonomic identifier in the sequence record from a local copy of the NCBI taxonomy database [48], which we downloaded from ftp://ftp.ncbi.nih.gov/pub/taxonomy/on March 2, 2011. See Methods S1 for additional details on the inferred taxonomic annotation of this training set.

For the ‘**filtered NCBI**’ training set we inferred the taxonomic classification, including species, using the NCBI taxonomic identifier in the sequence record and the local copy of the NCBI taxonomy database [48], utilizing Bio::LITE::Taxonomy::NCBI version 0.06. We removed from this dataset any sequence for which phylum or genus annotation was missing. We then performed additional filtering retaining only sequences whose classification in NCBI was consistent with their RDP annotation for phylum, class, order, family and genus or if the sequence classification contains a value unique to NCBI where there is disagreement between the databases (because this likely represents a recent addition to the taxonomic hierarchy). While performing this filtration we found systematic differences between the NCBI and RDP hierarchies. We added the sequences conforming with the filtration criteria after accounting for these systematic differences to the final training set. Finally, the ‘**LTP**’ training set consists of the bacterial sequences and taxonomic annotation from “The All-Species Living Tree" project (LTP) [41], [42]. We downloaded LTP release 106 annotation data and multiple sequence alignment from http://www.arb-silva.de/projects/living-tree/) on August 30, 2011. We used the “tax_ltp” field for taxonomic annotation down to the genus level, excluding annotations where the rank was specified as ‘Unclassified’. We obtained species names from the “fullname_ltp” field, removing subspecies information where present.

To facilitate the extraction of rank information from predicted taxonomic paths, we prefixed each taxon name within the training taxonomies with the corresponding rank. We also excluded taxonomic path components that began with “unclassified” or ended with “incertae_sedis”.

We tested the performance of different training sets by classifying sequences of simulated amplicons surrounding the V4 hypervariable region, which has been widely used for high-throughput 16S rRNA gene sequencing [10]. We focused on the 100 nt single-end sequencing strategy (namely, the first 100 bp of the amplicon), which is the most challenging experimental configuration among those examined in this work.

### Test Sets

To measure precision (or the false prediction rate, which is 1-[precision]) we used the bacterial sequences and taxonomic annotation from “The All-Species Living Tree" project (LTP) (Munoz, et al., 2011; Yarza, et al., 2008) for leave-k-out testing – this set is identical to the LTP training set. In turn, to measure coverage, we used 1,462,503 high quality 16S rRNA gene sequences amplified from environmental (uncultured) bacteria. We downloaded these sequences, in MSA format, from the RDP browser (http://rdp.cme.msu.edu/hierarchy/hb_intro.jsp, (Cole, et al., 2007; Cole, et al., 2009)) on September 21, 2011.

### Extraction of Amplicon Sequences

The basis for our analyses are the sequence reads that would have been obtained if the primer pairs (Table S1) produced an amplicon from any bacterial 16S rRNA gene sequence (namely, with no bias). To simulate this set of reads we started by extracting, for each primer pair, the subsequences it would amplify (i.e. the amplicons) from all available sequences that covered this region. This was performed for the LTP bacterial sequence set, the RDP bacterial isolates set, and the RDP environmental (uncultured) bacteria set, using python scripts that utilize Biopython [49] modules. First, we found the coordinates covered by the two primers on a reference sequence (Table S2). To avoid missing insertions that are adjacent to the primers we added one flanking position on each side of the amplicon. We then extracted the corresponding sub-alignment, including only sequences that begin before or at the first column of the sub-alignment and end after or at its last column. Finally, to obtain the collection of amplicons, we removed the first and last columns of this sub-alignment and the gap characters. To ensure the high quality of the RDP [43], [44] sequences included in our training sets we performed length filtration of the amplicons, which resulted in loss of at most 0.16% of sequences (see Methods S1).

### Generation of Short Read Data

To obtain single reads of length X bases (X = 100 or 120) from the amplicon data we extracted the first (forward read) or last (reverse read) X bases from each amplicon sequence. The read direction used (Table S1) was selected to optimize the number of hypervariable region bases covered. To obtain paired-end sequences for reads of length X from the amplicon data we fused the first and last X bases of the amplicon sequence, separating the two subsequences by a run of ten N’s. If the amplicon length was shorter or equal to 2X we used the amplicon sequence. Finally, we filtered out any sequence ‘reads’ that contained more than ten ambiguous characters (excluding intervening runs of N’s) from the ‘unfiltered RDP’ and ‘filtered NCBI’ training sets.

### Leave k Out Classification Tests

We evaluated the precision expected from each of the training sets by classifying sequence fragments derived from the bacterial portion of LTP [41], [42] using a leave-k-out approach. To classify a sequence S we first excluded from the training set all sequences annotated as derived from the same species. We then classified S, using the naïve Bayesian classifier implemented by the classify.seqs function in mothur v.1.20.1 [36] with 1000 bootstrap iterations. In all cases we trimmed the training taxonomies to contain information only down to the genus level (the highest resolution level evaluated).

We used the following procedure for the leave-k-out tests when training with the ‘RDP TS6’ or with the LTP training sets. First, we prepared an exclusion table, which related test (LTP) sequences to same-species training sequences. This process also produced a list of LTP sequences whose species were absent from the training set to begin with. Since these sequences could be tested without modifying the training set we classified them as one batch. For the remaining sequences, we iterated over the exclusion table, each time excluding the sequences belonging to the same species as the test sequence from the training set, and then using this modified training set to classify the test sequence.

The training sets derived from the 16S rRNA gene sequences of bacterial isolates downloaded from the RDP database [43], [44] were highly redundant. For tests using these training sets we used a modified procedure that minimizes the redundancy in sequence, whilst maximizing the taxonomic information available for training. To do so, we extracted from the full training set a subset of sequences that was non-redundant at the genus level. To this end, we used the USEARCH software [50] to cluster the sequences at the 100% identity level, using the ‘–iddef 1′ option that considers every mismatch and gap column to be a difference to define identity. We then used the resulting clusters file, as well as the taxonomic annotation for the sequences, to obtain sequence files in which two identical sequences were retained only if they belonged to different genera. This reduced set will subsequently be referred to as the *‘* ***original non-redundant training set*** *’*. This set was used for classifying the test sequences whose species were absent from it.

For the remaining species we generated an exclusion table from the full set of sequences, as described above. Then, for each test sequence in the exclusion table, we removed the same-species sequences from the full training set, and subsequently used the USEARCH [50] cluster file generated previously with the taxonomic annotation, to create a *non-redundant training set* from the remaining sequences, which was then used to classify the test-sequence. This avoided the potential elimination of available same-genus different-species sequences from the training set, which would make the test stricter than intended. For examining the effect of classifying 100 nt single-reads with a training set trimmed down to the same 100 nt region or to the complete amplicon (Figures S3, S4, S5), we removed redundancy using only the 100 nt region tested for both the trimming regimes. Thus, we could focus on the effect of additional bases within the same sequence without the confounding effects of additional sequences. Similar results were obtained when we used the complete amplicon sequence to filter redundancy for both the 100 nt training set and the complete amplicon training set.

### Classification of Environmental Sequences

We used the naïve Bayesian classifier implemented by the classify.seqs function in mothur v.1.20.1 [36] with 1000 bootstrap iterations to classify the sequence reads derived from environmental (uncultured) bacteria (see Table S3, for the number of reads covering each region examined), each time training with a different training set (Table 1). This classifier implements the same algorithm as the RDP classifier [23] and performs equivalently (see Methods S1). For training sets in which redundancy was removed for the leave-k-out testing, we used the ‘original non-redundant training set’ for training (see Methods section ‘Leave k out classification tests’). For consistency with the leave-k-out tests, we used the rearranged taxonomies for all training sets (see Methods S1).

### Combining Classifications from Two Regions

To combine the predictions obtained from regions *A* and *B* of the same 16S rRNA gene molecule we examined the prediction for rank *R*, the highest resolution rank predicted by both classifications (in most cases this was genus). We then copied the entire taxonomic path from the prediction with the highest confidence score for rank *R* to the combined classification file. Sequences with equal confidence but different predictions at rank *R* were discarded (<1% of sequences in any dataset). We included only sequences covering both region *A* and region *B* in the set of combined predictions.

### Statistical Analyses

To evaluate the predictions made in the leave-k-out tests we compared the prediction to the corresponding reference (LTP) annotation, evaluating each taxonomic rank separately. A prediction at rank *R* was defined as true if the predicted and reference annotations matched or were synonymous. Conversely, a false prediction at rank *R* was defined as a case where both the predicted and reference annotations included this rank, but the taxon names did not match, or the rank was included only in the prediction (implying that the sequence does not belong in any of the currently defined taxa at the relevant rank). The false prediction rate (FPR) was defined as.
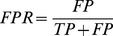
(1)Here, *TP* is the number of true predictions, and *FP* is the number of false predictions.

To compare the performance across training sets and regions, as well as formulate recommendations, we used a set, *E,* of FPR values ranging from 0.001 to 0.95. For each *e*∈*E*, we determined a conservative confidence threshold that would ensure that FPR would be at most *e*, irrespective of the distribution of confidence values. Specifically, we computed *TP* and *FP* counts over confidence score intervals of size 5 (0–4, …, 95–99), except for confidence score 100, which we calculated using an interval of size 1. Then, using the binomial distribution, we determined for each interval the probability that at most *TP* true predictions would be observed assuming *FPR = e*. Finally, going down the set of intervals starting at confidence score 100, we looked for the first interval for which this probability was at most 0.05, and picked the interval above it as the threshold. Thus, the chosen confidence threshold conservatively assumes that all confidence scores fall into the threshold bin. For added robustness, each leave-k-out test was repeated 100 times and for each *e*∈*E* the final threshold was chosen as the median of thresholds obtained for *e* in the repeats. Tables S4, S5, S6, S7, S8, S9, and S10 contain, for all regions and sequencing strategies, the coverage in environmental sequences (see below) including both the cumulative and interval TP, FP, precision and FPR. For confidence scores that were selected as cutoffs, the corresponding desired FPR is also indicated.

We define the coverage for rank *R* and confidence threshold *c* (0≤*c*≤100) as follows:
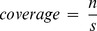
(2)Here, *n* is the number of times *R* is predicted with confidence greater or equal to *c*, and *S* is the total number of sequences with a prediction at any level, including domain (the fraction of sequences for which we failed to get any classification was at most 6e-05).

The taxonomic hierarchies underlying the training sets contain gaps. In the RDP hierarchy, for example, there are seven phyla and three classes that have no child taxa, and in the phylum Acidobacteria only classes and genera are defined, omitting the ranks order and family. These features of the hierarchy could lead to under-estimation of prediction rates, affecting some ranks more than others. We corrected the prediction rate for rank *R* by computing, post-hoc, the difference between the total number of environmental sequences and the number of sequences classified to places in the training hierarchy that omit rank *R*, and using this number as the denominator in the calculation of coverage. Throughout the paper we used the corrected values of coverage.

For a fair comparison of different regions, we included only the 8,394 sequences covering the V3–V7 region for the calculation of FPR. Similarly, to maximize the overlap in the set used to calculate coverage, we included only the 854,766 environmental sequences covering regions V3–V6. See Table S3 for the coverage of each of the regions by the LTP and RDP environmental sequences.

All plots were generated with the ggplot2 package [51]. For each desired FPR we calculated the coverage of environmental sequences obtained using the corresponding confidence threshold. We then plotted desired FPR against coverage, ending each plot at the point where a confidence threshold of 0 was reached.

### Microbiome Sample Preparation

The microbiome samples used were derived from stool collected from two healthy adults during February 2011. Stool was immediately frozen after collection at −20°C until permanent storage at −80°C. 0.25g frozen stool was used for DNA extraction with the Omega Bio-Tek E.Z.N.A. Stool DNA Kit (Omega Bio-Tek, USA), following provided instructions (revisions March 2010). DNA concentration and purity were assessed using the Nanodrop 1000 spectrophotometer (Thermo Scientific, USA). The V4 region was amplified using the protocol published by Caporaso et al. [10] with the following adjustments: 3×50 ng starting template reactions were combined per sample and samples were quality controlled at the end of library preparation using the Agilent Bioanalyzer DNA 1000 kit (Agilent Technologies, USA). Samples were sequenced on an Illumina HiSeq2000 and data pre-processed with CASAVA 1.8.1 (Illumina, USA). Reads were quality controlled using the procedure by Caporaso et al with the following alterations: 1. The beginning of the read was truncated at the point where it incurred two adjacent low-quality base calls within the first 13 base pairs and 2. low quality was considered Q20 or below. The sequence data have been deposited in the National Center for Biotechnology Information short read archive (http://www.ncbi.nlm.nih.gov/Traces/sra/sra.cgi). Accession numbers to come.

## Supporting Information

Figure S1 **Distribution of confidence scores obtained in leave-k-out tests.** We classified 100 nt single reads from the V4 region using four different training sets. Each panel shows the distribution of confidence scores for a different rank. We counted the number of predictions using confidence score bins 0–4,5–9,…,95–99, 100. Each point is the median count obtained from 100 repeats of the leave-k-out test.
(TIF)

Figure S2 **Performance of different sequence trimming regimes of the LTP training set.** We used the LTP training set to classify 100 nt reads from the V4 amplicon, utilizing two alternative trimming regimes to the training set sequences - first 100 nt vs. complete amplicon. Each panel compares the performance of the trimming regimes for a different rank. We used the results of leave-k-out tests classifying the LTP sequences to determine confidence score thresholds for a set of desired false prediction rate (FPR) values (x axis), so that the FPR would be at most the desired value. We then used these thresholds to calculate the classification coverage of sequences from environmental (uncultured) bacteria that corresponds to the desired FPR (y axis).
(TIF)

Figure S3 **Performance of different sequence trimming regimes of the ‘unfiltered RDP’ training set**. We used the ‘unfiltered RDP’ training set to classify 100 nt reads from the V4 amplicon, utilizing two alternative trimming regimes to the training set sequences - first 100 nt vs. complete amplicon. Each panel compares the performance of the trimming regimes for a different rank. Because for this training set we removed redundancy, two regimes of removing redundancy are examined for each trimming regime – using the first 100 bp of the amplicon or the complete amplicon. We used the results of leave-k-out tests classifying the LTP sequences to determine confidence score thresholds for a set of desired false prediction rate (FPR) values (x axis), so that the FPR would be at most the desired value. We then used these thresholds to calculate the classification coverage of sequences from environmental (uncultured) bacteria that corresponds to the desired FPR (y axis).
(TIF)

Figure S4 **Performance of different sequence trimming regimes of the ‘filtered NCBI’ training set.** We used the ‘unfiltered RDP’ training set to classify 100 nt reads from the V4 amplicon, utilizing two alternative trimming regimes to the training set sequences - first 100 nt vs. complete amplicon. Each panel compares the performance of the trimming regimes for a different rank. Because for this training set we removed redundancy, two regimes of removing redundancy are examined for each trimming regime – using the first 100 bp of the amplicon or the complete amplicon. We used the results of leave-k-out tests classifying the LTP sequences to determine confidence score thresholds for a set of desired false prediction rate (FPR) values (x axis), so that the FPR would be at most the desired value. We then used these thresholds to calculate the classification coverage of sequences from environmental (uncultured) bacteria that corresponds to the desired FPR (y axis).
(TIF)

Figure S5 **Performance of different experimental designs in classifying ranks order to phylum.** Each panel compares performance of different regions for a different combination of rank (order, class, or phylum) and sequencing strategy (100/120 nt single/paired-end reads). We used the results of leave-k-out tests classifying the LTP sequences to determine confidence score thresholds for a set of desired false prediction rate (FPR) values (x axis), so that the FPR would be at most the desired value (Tables S4, S5, S6, S7, S8, S9 and S10). We then used these thresholds to calculate the classification coverage of sequences from environmental (uncultured) bacteria that corresponds to the desired FPR (y axis). Note the variation in x-axis ranges among the different ranks.
(TIF)

Figure S6 **Performance of combined 100** **nt single-read predictions in classifying ranks order to phylum.** We combined predictions made for different 100 nt fragments of the same sequence, by selecting the prediction with the highest confidence score at the genus level (or the lowest common level available). We evaluated the performance of combinations of fragments from the V3 and V4 regions (top and bottom panels, respectively) with fragments from each of the other regions examined, and compared it to the performance of the V3 (orange curve in top panels) and V4 (light blue curve in bottom panels) 100 nt paired-end configurations. We used the results of leave-k-out tests classifying the LTP sequences to determine confidence score thresholds for a set of desired false prediction rate (FPR) values (x axis), so that the FPR would be at most the desired value. We then used these thresholds to calculate the classification coverage of sequences from environmental (uncultured) bacteria that corresponds to the desired FPR (y axis). Note the variation in x-axis ranges among the different ranks.
(TIF)

Table S1 **Primer pairs studied.** The sequences of the primers in each pair, the read direction used for single-read sequencing, and the hypervariable region/s of the 16S rRNA gene covered using the sequencing strategies studied in this work are indicated.
(DOC)

Table S2 **Coordinates of the amplicons studied on the reference sequences used**: RDP database bacterial isolates – S000495522 (GenBank AB035921); LTP – X80725 (*) and AJ508775 (**); RDP uncultured bacteria – S001235409 (Genbank FJ479556).
(DOC)

Table S3 **Coverage of the examined amplicons by LTP and RDP uncultured bacterial sequences.** We identify each amplicon using the 16S rRNA gene hypervariable region covered by single-read sequencing configurations. We counted sequences only if they contained the entire amplicon.
(DOC)

Table S4 **Summary of analyses for V1.** For each sequencing configuration, taxonomic rank and bootstrap confidence value C, we provide the following information pertaining to the interval [C.C+4] (except for the case C = 100, in which the interval is of size 1): Interval_TP - median number of true predictions in leave-k-out tests (out of 100 repeats); Interval_FP - median number of false predictions in leave-k-out tests (out of 100 repeats); Interval_precision - precision in leave-k-out tests (%), calculated from Interval_TP and Interval_FP; Interval_FPR - false prediction rate in leave-k-out tests (%), calculated from Interval_TP and Interval_FP; threshold for FPR – FPR values for which the confidence value is suitable as a threshold (note that not all rows will contain this field, as we tested only a limited set of FPR values), each threshold is the median of 100 thresholds obtained for the same desired FPR from 100 repeats of the leave-k-out test. We also provide the following information for the interval [C.100]: Env_coverage - coverage of environmental sequences (%); Cumulative_TP - number of true predictions in leave-k-out tests, calculated from Interval_TP; Cumulative_FP - number of false predictions in leave-k-out tests, calculated from Interval_FP; Cumulative_precision – precision of leave-k-out tests (%), calculated from Cumulative_TP and Cumulative_FP; Cumulative_FPR – false prediction rate of leave-k-out tests (%), calculated from Cumulative_TP and Cumulative_FP.
(XLS)

Table S5 **Summary of analyses for V3.** For each sequencing configuration, taxonomic rank and bootstrap confidence value C, we provide the following information pertaining to the interval [C.C+4] (except for the case C = 100, in which the interval is of size 1): Interval_TP - median number of true predictions in leave-k-out tests (out of 100 repeats); Interval_FP - median number of false predictions in leave-k-out tests (out of 100 repeats); Interval_precision - precision in leave-k-out tests (%), calculated from Interval_TP and Interval_FP; Interval_FPR - false prediction rate in leave-k-out tests (%), calculated from Interval_TP and Interval_FP; threshold for FPR – FPR values for which the confidence value is suitable as a threshold (note that not all rows will contain this field, as we tested only a limited set of FPR values), each threshold is the median of 100 thresholds obtained for the same desired FPR from 100 repeats of the leave-k-out test. We also provide the following information for the interval [C.100]: Env_coverage - coverage of environmental sequences (%); Cumulative_TP - number of true predictions in leave-k-out tests, calculated from Interval_TP; Cumulative_FP - number of false predictions in leave-k-out tests, calculated from Interval_FP; Cumulative_precision – precision of leave-k-out tests (%), calculated from Cumulative_TP and Cumulative_FP; Cumulative_FPR – false prediction rate of leave-k-out tests (%), calculated from Cumulative_TP and Cumulative_FP.
(XLS)

Table S6 **Summary of analyses for V4.** For each sequencing configuration, taxonomic rank and bootstrap confidence value C, we provide the following information pertaining to the interval [C.C+4] (except for the case C = 100, in which the interval is of size 1): Interval_TP - median number of true predictions in leave-k-out tests (out of 100 repeats); Interval_FP - median number of false predictions in leave-k-out tests (out of 100 repeats); Interval_precision - precision in leave-k-out tests (%), calculated from Interval_TP and Interval_FP; Interval_FPR - false prediction rate in leave-k-out tests (%), calculated from Interval_TP and Interval_FP; threshold for FPR – FPR values for which the confidence value is suitable as a threshold (note that not all rows will contain this field, as we tested only a limited set of FPR values), each threshold is the median of 100 thresholds obtained for the same desired FPR from 100 repeats of the leave-k-out test. We also provide the following information for the interval [C.100]: Env_coverage - coverage of environmental sequences (%); Cumulative_TP - number of true predictions in leave-k-out tests, calculated from Interval_TP; Cumulative_FP - number of false predictions in leave-k-out tests, calculated from Interval_FP; Cumulative_precision – precision of leave-k-out tests (%), calculated from Cumulative_TP and Cumulative_FP; Cumulative_FPR – false prediction rate of leave-k-out tests (%), calculated from Cumulative_TP and Cumulative_FP.
(XLS)

Table S7 **Summary of analyses for V5.** For each sequencing configuration, taxonomic rank and bootstrap confidence value C, we provide the following information pertaining to the interval [C.C+4] (except for the case C = 100, in which the interval is of size 1): Interval_TP - median number of true predictions in leave-k-out tests (out of 100 repeats); Interval_FP - median number of false predictions in leave-k-out tests (out of 100 repeats); Interval_precision - precision in leave-k-out tests (%), calculated from Interval_TP and Interval_FP; Interval_FPR - false prediction rate in leave-k-out tests (%), calculated from Interval_TP and Interval_FP; threshold for FPR – FPR values for which the confidence value is suitable as a threshold (note that not all rows will contain this field, as we tested only a limited set of FPR values), each threshold is the median of 100 thresholds obtained for the same desired FPR from 100 repeats of the leave-k-out test. We also provide the following information for the interval [C.100]: Env_coverage - coverage of environmental sequences (%); Cumulative_TP - number of true predictions in leave-k-out tests, calculated from Interval_TP; Cumulative_FP - number of false predictions in leave-k-out tests, calculated from Interval_FP; Cumulative_precision – precision of leave-k-out tests (%), calculated from Cumulative_TP and Cumulative_FP; Cumulative_FPR – false prediction rate of leave-k-out tests (%), calculated from Cumulative_TP and Cumulative_FP.
(XLS)

Table S8 **Summary of analyses for V6.** For each sequencing configuration, taxonomic rank and bootstrap confidence value C, we provide the following information pertaining to the interval [C.C+4] (except for the case C = 100, in which the interval is of size 1): Interval_TP - median number of true predictions in leave-k-out tests (out of 100 repeats); Interval_FP - median number of false predictions in leave-k-out tests (out of 100 repeats); Interval_precision - precision in leave-k-out tests (%), calculated from Interval_TP and Interval_FP; Interval_FPR - false prediction rate in leave-k-out tests (%), calculated from Interval_TP and Interval_FP; threshold for FPR – FPR values for which the confidence value is suitable as a threshold (note that not all rows will contain this field, as we tested only a limited set of FPR values), each threshold is the median of 100 thresholds obtained for the same desired FPR from 100 repeats of the leave-k-out test. We also provide the following information for the interval [C.100]: Env_coverage - coverage of environmental sequences (%); Cumulative_TP - number of true predictions in leave-k-out tests, calculated from Interval_TP; Cumulative_FP - number of false predictions in leave-k-out tests, calculated from Interval_FP; Cumulative_precision – precision of leave-k-out tests (%), calculated from Cumulative_TP and Cumulative_FP; Cumulative_FPR – false prediction rate of leave-k-out tests (%), calculated from Cumulative_TP and Cumulative_FP.
(XLS)

Table S9 **Summary of analyses for V7.** For each sequencing configuration, taxonomic rank and bootstrap confidence value C, we provide the following information pertaining to the interval [C.C+4] (except for the case C = 100, in which the interval is of size 1): Interval_TP - median number of true predictions in leave-k-out tests (out of 100 repeats); Interval_FP - median number of false predictions in leave-k-out tests (out of 100 repeats); Interval_precision - precision in leave-k-out tests (%), calculated from Interval_TP and Interval_FP; Interval_FPR - false prediction rate in leave-k-out tests (%), calculated from Interval_TP and Interval_FP; threshold for FPR – FPR values for which the confidence value is suitable as a threshold (note that not all rows will contain this field, as we tested only a limited set of FPR values), each threshold is the median of 100 thresholds obtained for the same desired FPR from 100 repeats of the leave-k-out test. We also provide the following information for the interval [C.100]: Env_coverage - coverage of environmental sequences (%); Cumulative_TP - number of true predictions in leave-k-out tests, calculated from Interval_TP; Cumulative_FP - number of false predictions in leave-k-out tests, calculated from Interval_FP; Cumulative_precision – precision of leave-k-out tests (%), calculated from Cumulative_TP and Cumulative_FP; Cumulative_FPR – false prediction rate of leave-k-out tests (%), calculated from Cumulative_TP and Cumulative_FP.
(XLS)

Table S10 **Summary of analyses for V9.** For each sequencing configuration, taxonomic rank and bootstrap confidence value C, we provide the following information pertaining to the interval [C.C+4] (except for the case C = 100, in which the interval is of size 1): Interval_TP - median number of true predictions in leave-k-out tests (out of 100 repeats); Interval_FP - median number of false predictions in leave-k-out tests (out of 100 repeats); Interval_precision - precision in leave-k-out tests (%), calculated from Interval_TP and Interval_FP; Interval_FPR - false prediction rate in leave-k-out tests (%), calculated from Interval_TP and Interval_FP; threshold for FPR – FPR values for which the confidence value is suitable as a threshold (note that not all rows will contain this field, as we tested only a limited set of FPR values), each threshold is the median of 100 thresholds obtained for the same desired FPR from 100 repeats of the leave-k-out test. We also provide the following information for the interval [C.100]: Env_coverage - coverage of environmental sequences (%); Cumulative_TP - number of true predictions in leave-k-out tests, calculated from Interval_TP; Cumulative_FP - number of false predictions in leave-k-out tests, calculated from Interval_FP; Cumulative_precision – precision of leave-k-out tests (%), calculated from Cumulative_TP and Cumulative_FP; Cumulative_FPR – false prediction rate of leave-k-out tests (%), calculated from Cumulative_TP and Cumulative_FP.
(XLS)

Methods S1 (DOC)
