# Supplementary figures and images for: Taxonomic Classification of Bacterial 16S rRNA Genes Using Short Sequencing Reads: Evaluation of Effective Study Designs
Source: PLoS One. 2013 Jan 7;8(1):e53608. doi: 10.1371/journal.pone.0053608 (PMC3538547; doi:10.1371/journal.pone.0053608)

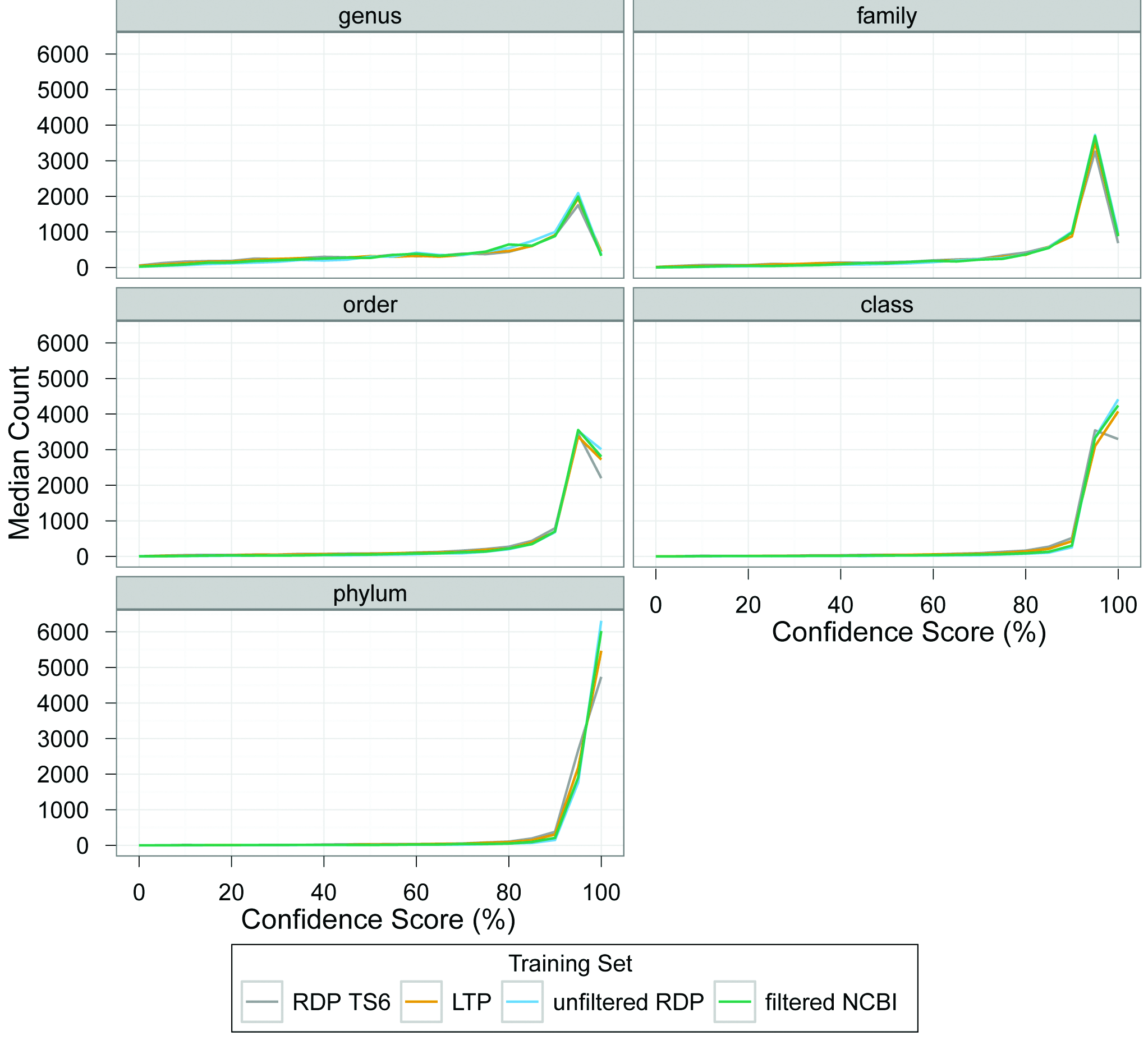

Supplement: Figure S1 — Distribution of confidence scores obtained in leave-k-out tests. We classified 100 nt single reads from the V4 region using four different training sets. Each panel shows the distribution of confidence scores for a different rank. We counted the number of predictions using confidence score bins 0–4,5–9,…,95–99, 100. Each point is the median count obtained from 100 repeats of the leave-k-out test. (TIF) [file pone.0053608.s001.tif]

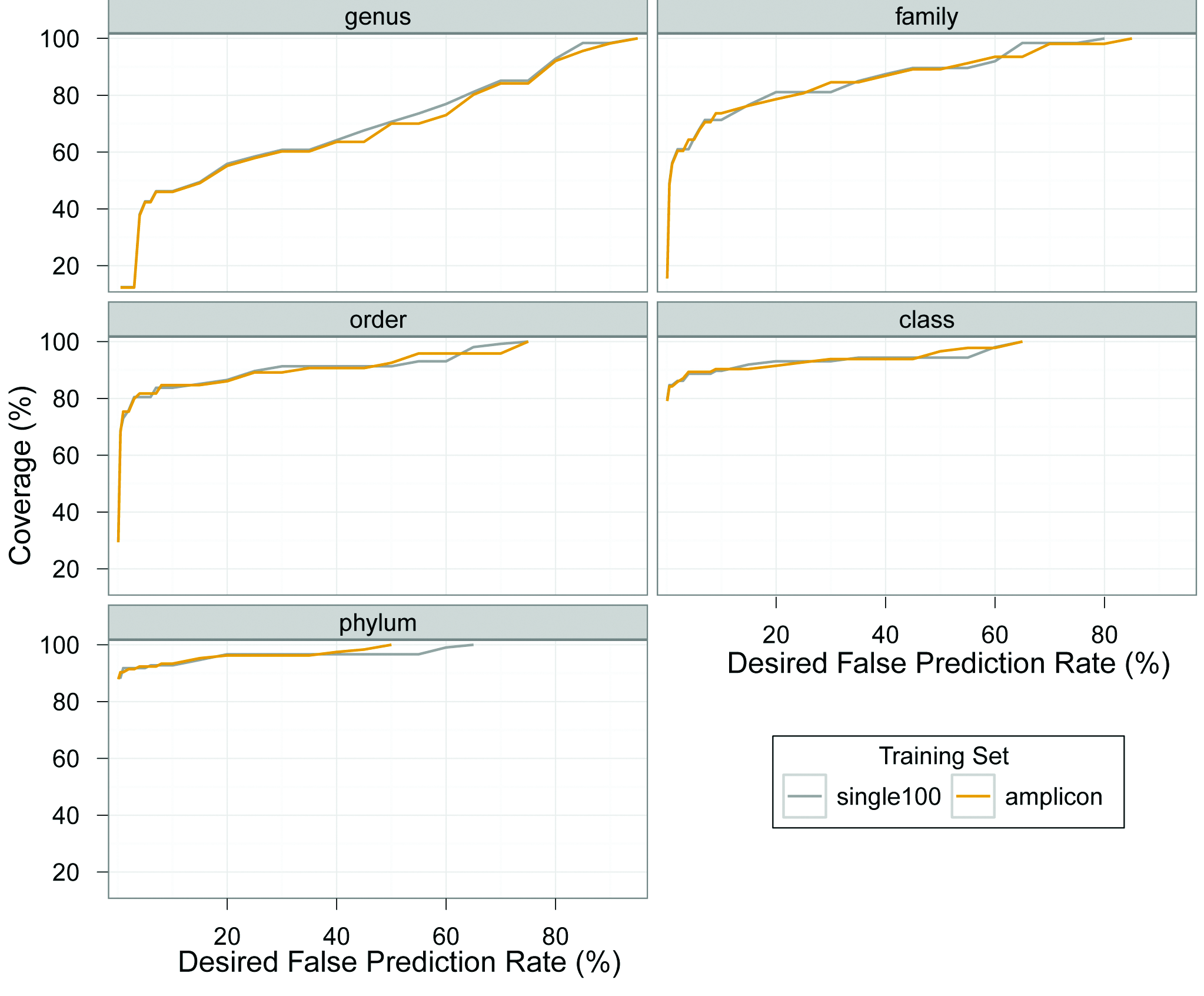

Supplement: Figure S2 — Performance of different sequence trimming regimes of the LTP training set. We used the LTP training set to classify 100 nt reads from the V4 amplicon, utilizing two alternative trimming regimes to the training set sequences - first 100 nt vs. complete amplicon. Each panel compares the performance of the trimming regimes for a different rank. We used the results of leave-k-out tests classifying the LTP sequences to determine confidence score thresholds for a set of desired false prediction rate (FPR) values (x axis), so that the FPR would be at most the desired value. We then used these thresholds to calculate the classification coverage of sequences from environmental (uncultured) bacteria that corresponds to the desired FPR (y axis). (TIF) [file pone.0053608.s002.tif]

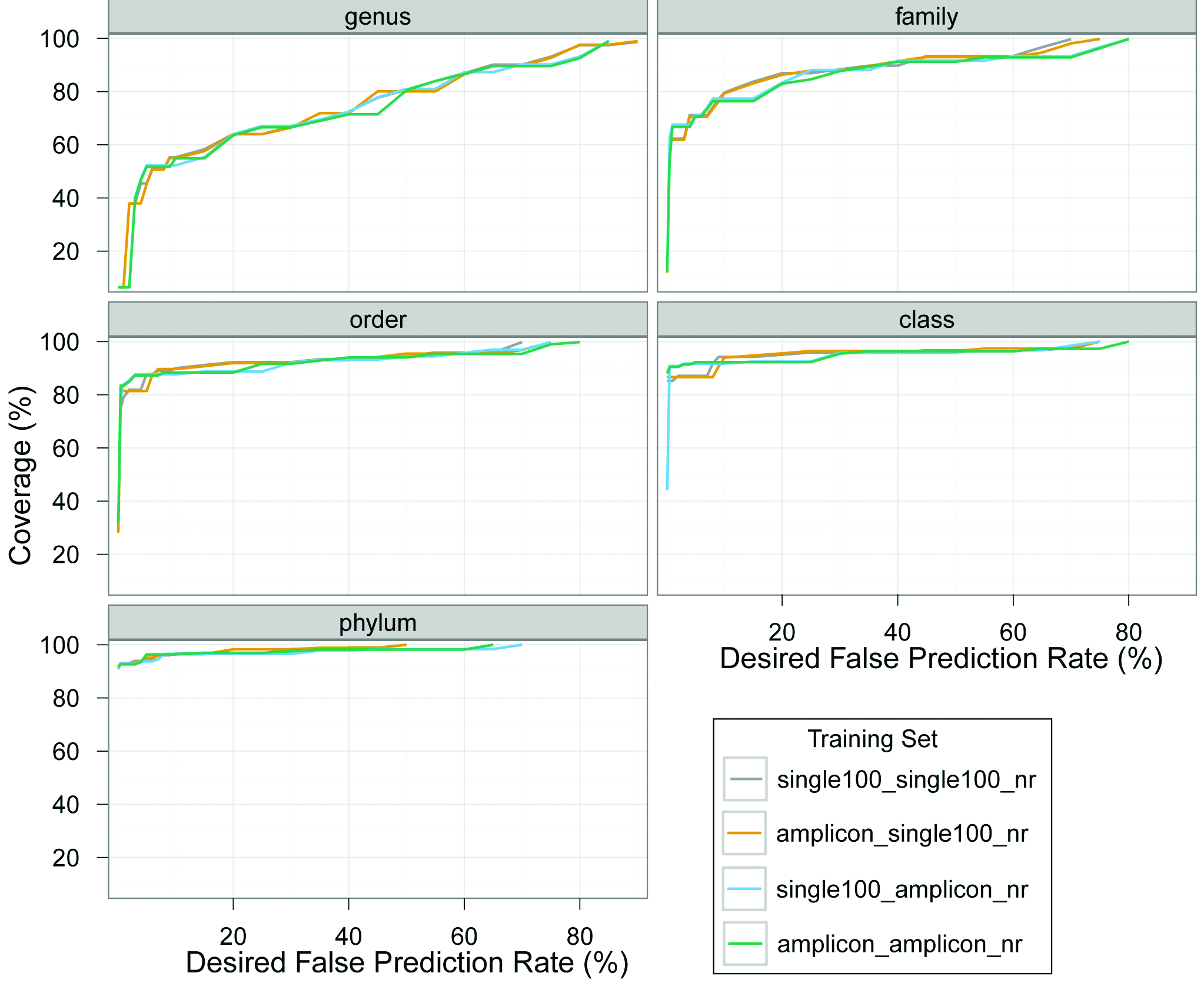

Supplement: Figure S3 — Performance of different sequence trimming regimes of the ‘unfiltered RDP’ training set. We used the ‘unfiltered RDP’ training set to classify 100 nt reads from the V4 amplicon, utilizing two alternative trimming regimes to the training set sequences - first 100 nt vs. complete amplicon. Each panel compares the performance of the trimming regimes for a different rank. Because for this training set we removed redundancy, two regimes of removing redundancy are examined for each trimming regime – using the first 100 bp of the amplicon or the complete amplicon. We used the results of leave-k-out tests classifying the LTP sequences to determine confidence score thresholds for a set of desired false prediction rate (FPR) values (x axis), so that the FPR would be at most the desired value. We then used these thresholds to calculate the classification coverage of sequences from environmental (uncultured) bacteria that corresponds to the desired FPR (y axis). (TIF) [file pone.0053608.s003.tif]

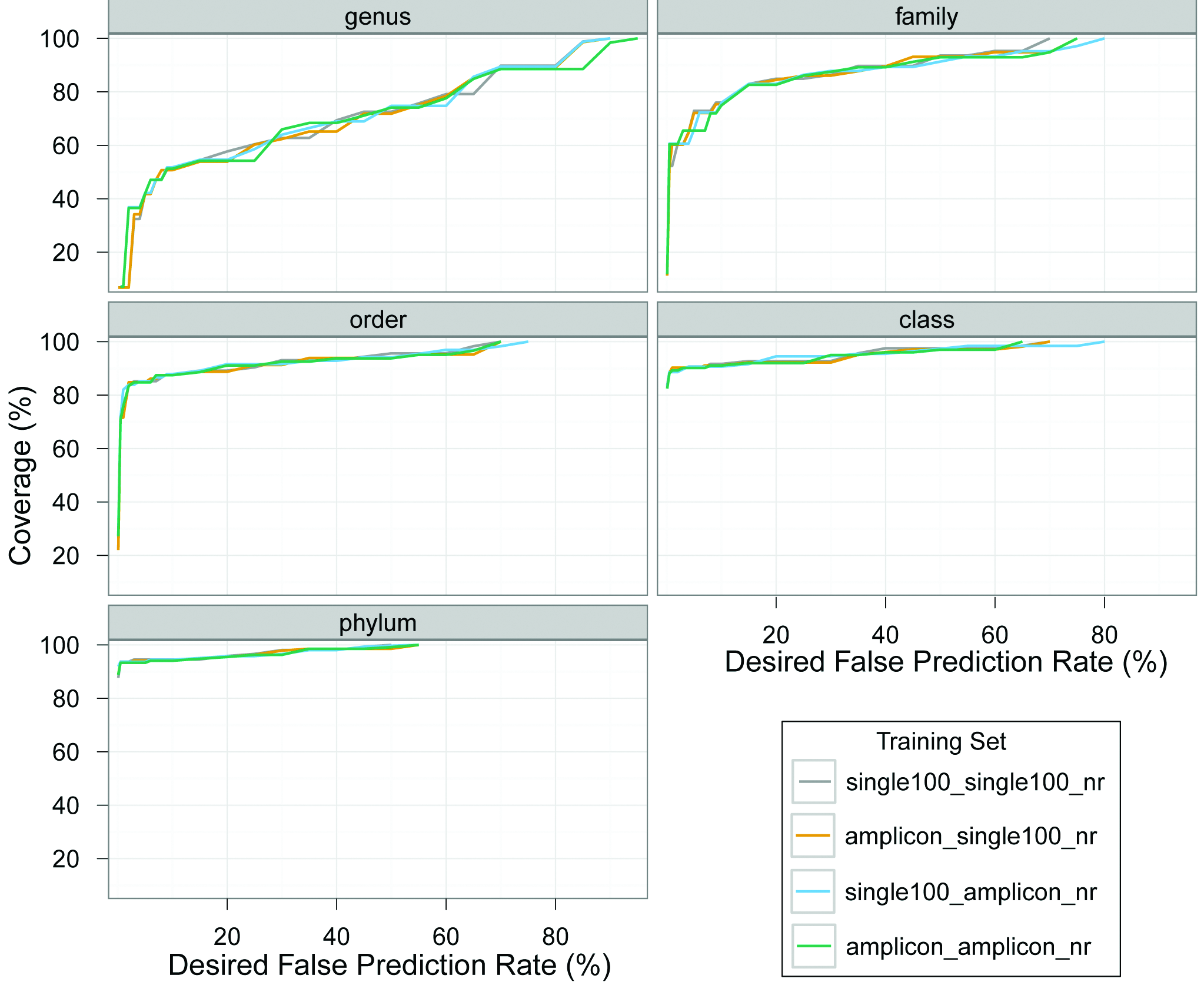

Supplement: Figure S4 — Performance of different sequence trimming regimes of the ‘filtered NCBI’ training set. We used the ‘unfiltered RDP’ training set to classify 100 nt reads from the V4 amplicon, utilizing two alternative trimming regimes to the training set sequences - first 100 nt vs. complete amplicon. Each panel compares the performance of the trimming regimes for a different rank. Because for this training set we removed redundancy, two regimes of removing redundancy are examined for each trimming regime – using the first 100 bp of the amplicon or the complete amplicon. We used the results of leave-k-out tests classifying the LTP sequences to determine confidence score thresholds for a set of desired false prediction rate (FPR) values (x axis), so that the FPR would be at most the desired value. We then used these thresholds to calculate the classification coverage of sequences from environmental (uncultured) bacteria that corresponds to the desired FPR (y axis). (TIF) [file pone.0053608.s004.tif]

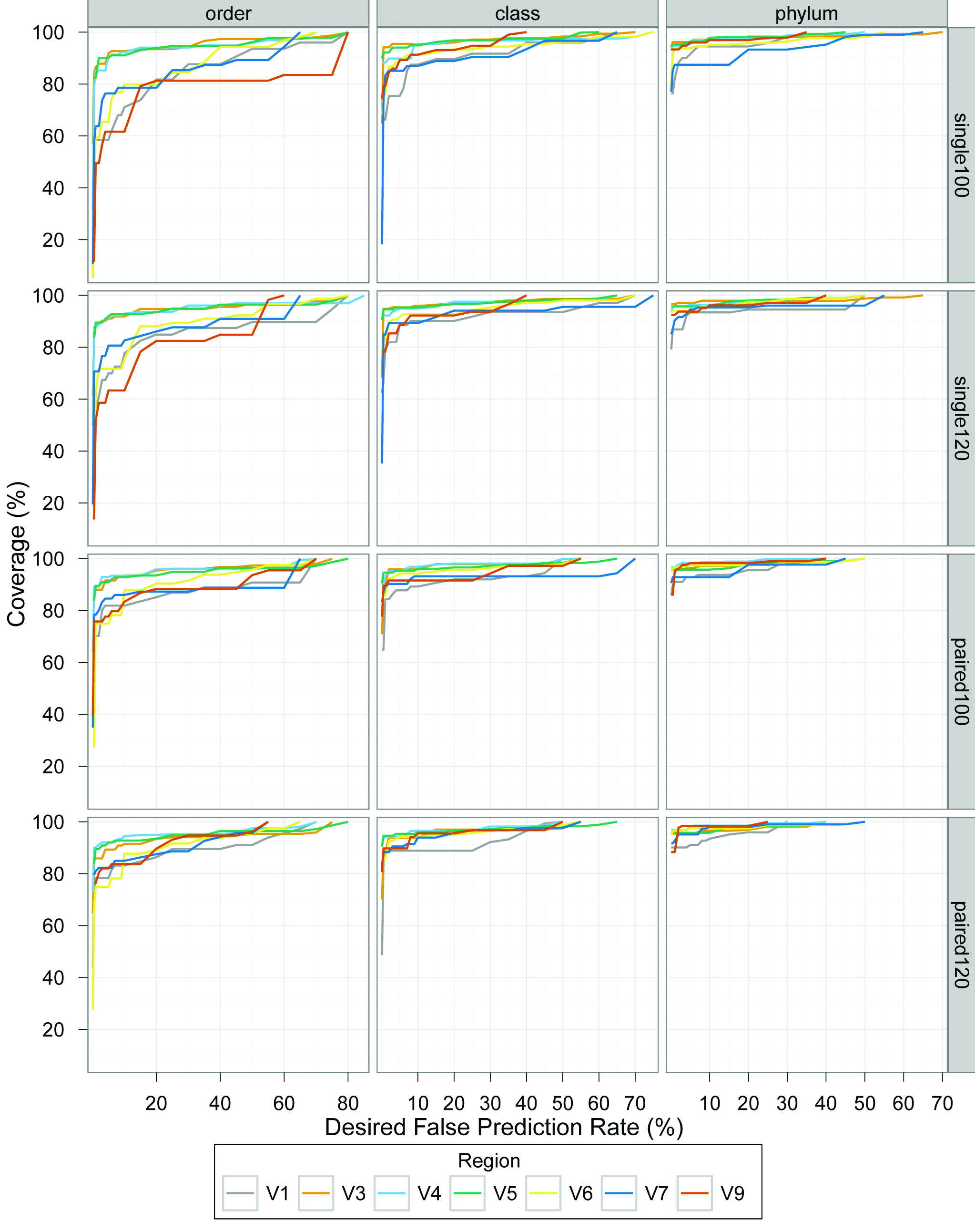

Supplement: Figure S5 — Performance of different experimental designs in classifying ranks order to phylum. Each panel compares performance of different regions for a different combination of rank (order, class, or phylum) and sequencing strategy (100/120 nt single/paired-end reads). We used the results of leave-k-out tests classifying the LTP sequences to determine confidence score thresholds for a set of desired false prediction rate (FPR) values (x axis), so that the FPR would be at most the desired value (Tables S4, S5, S6, S7, S8, S9 and S10). We then used these thresholds to calculate the classification coverage of sequences from environmental (uncultured) bacteria that corresponds to the desired FPR (y axis). Note the variation in x-axis ranges among the different ranks. (TIF) [file pone.0053608.s005.tif]

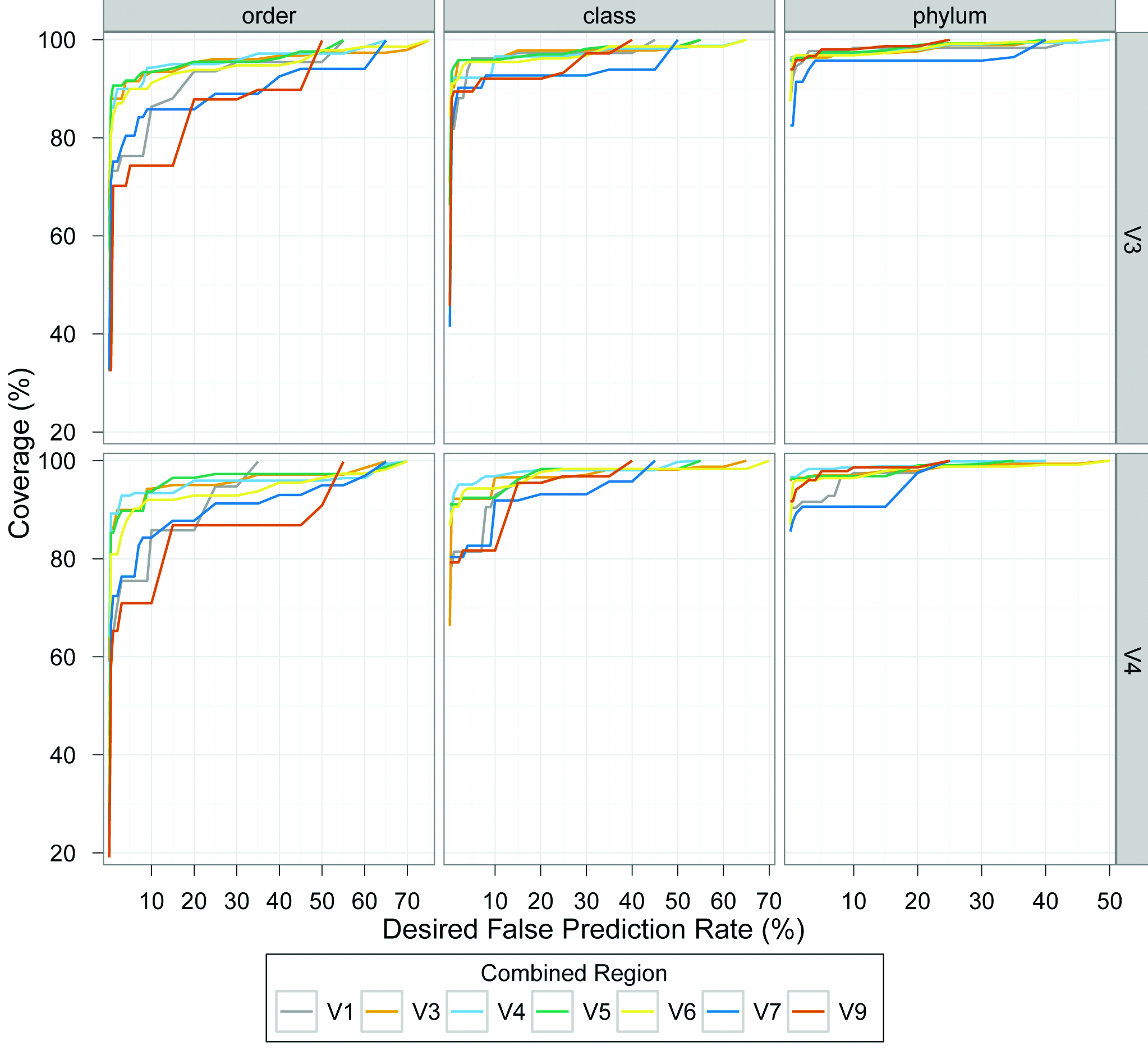

Supplement: Figure S6 — Performance of combined 100 nt single-read predictions in classifying ranks order to phylum. We combined predictions made for different 100 nt fragments of the same sequence, by selecting the prediction with the highest confidence score at the genus level (or the lowest common level available). We evaluated the performance of combinations of fragments from the V3 and V4 regions (top and bottom panels, respectively) with fragments from each of the other regions examined, and compared it to the performance of the V3 (orange curve in top panels) and V4 (light blue curve in bottom panels) 100 nt paired-end configurations. We used the results of leave-k-out tests classifying the LTP sequences to determine confidence score thresholds for a set of desired false prediction rate (FPR) values (x axis), so that the FPR would be at most the desired value. We then used these thresholds to calculate the classification coverage of sequences from environmental (uncultured) bacteria that corresponds to the desired FPR (y axis). Note the variation in x-axis ranges among the different ranks. (TIF) [file pone.0053608.s006.tif]
