# Supplementary material for: Taxonomic Classification of Bacterial 16S rRNA Genes Using Short Sequencing Reads: Evaluation of Effective Study Designs
Source: PLoS One. 2013 Jan 7;8(1):e53608. doi: 10.1371/journal.pone.0053608 (PMC3538547; doi:10.1371/journal.pone.0053608)
Supplement: Table S1 — Primer pairs studied. The sequences of the primers in each pair, the read direction used for single-read sequencing, and the hypervariable region/s of the 16S rRNA gene covered using the sequencing strategies studied in this work are indicated. (DOC) [file pone.0053608.s007.doc]

**Table S1. Primer pairs studied.**

| **Primer pair** | **Forward primer** | **Reverse primer** | **Region^a^** | **Single-read sequencing direction** | **References** |
| --- | --- | --- | --- | --- | --- |
| **27F+R357** | AGAGTTTGATCMTGGCTCAG | CTGCTGCCTYCCGTA | V1 | Forward |  |
| **F343+R534** | TACGGRAGGCAGCAG | ATTACCGCGGCTGCTGGC | V3 | Reverse |  |
| **F515+R806** | GTGCCAGCMGCCGCGGTAA | TAATCTWTGGGVHCATCAGG | V4 | Forward |  |
| **F784+R926** | RGGATTAGATACCCC | CCGTCAATTYYTTTRAGTTT | V5 | Forward |  |
| **F917+R1114** | GAATTGACGGGGRCCC | GGGTTGCGCTCGTTRC | V6 | Reverse |  |
| **F1099+R1391** | GYAACGAGCGCAACCC | GACGGGCGGTGTGTRCA | V7 | Forward |  |
| **F1099+R1492** | GYAACGAGCGCAACCC | GGTTACCTTGTTACGACTT | V7, V9 | Reverse |  |

^a^ Only hypervariable regions encompassed, at least in part, by any of the sequencing strategies studied are indicated.

References

1. Lane DJ (1991) 16S/23S rRNA sequencing. In: Stackebrandt E, Goodfellow M, editors. Nucleic acid techniques in bacterial systematics. New York, NY: John Wiley & Sons, Inc. pp. 115-175.

2. Liu Z, Lozupone C, Hamady M, Bushman FD, Knight R (2007) Short pyrosequencing reads suffice for accurate microbial community analysis. Nucleic Acids Res 35: e120.

3. Caporaso JG, Lauber CL, Walters WA, Berg-Lyons D, Lozupone CA, et al. (2011) Global patterns of 16S rRNA diversity at a depth of millions of sequences per sample. Proc Natl Acad Sci U S A 108 Suppl 1: 4516-4522.

4. Turner S, Pryer KM, Miao VP, Palmer JD (1999) Investigating deep phylogenetic relationships among cyanobacteria and plastids by small subunit rRNA sequence analysis. J Eukaryot Microbiol 46: 327-338.
