# Supplementary material for: Taxonomic Classification of Bacterial 16S rRNA Genes Using Short Sequencing Reads: Evaluation of Effective Study Designs
Source: PLoS One. 2013 Jan 7;8(1):e53608. doi: 10.1371/journal.pone.0053608 (PMC3538547; doi:10.1371/journal.pone.0053608)
Supplement: Table S2 — Coordinates of the amplicons studied on the reference sequences used: RDP database bacterial isolates – S000495522 (GenBank AB035921); LTP – X80725 (*) and AJ508775 (**); RDP uncultured bacteria – S001235409 (Genbank FJ479556). (DOC) [file pone.0053608.s008.doc]

**Table S2.** **Coordinates of the amplicons studied on the reference sequences used**: RDP database bacterial isolates – S000495522 (GenBank AB035921); LTP – X80725 (*) and AJ508775 (**); RDP uncultured bacteria – S001235409 (Genbank FJ479556).

| **Primer combination** | **Amplicon coordinates on RDP bacterial isolates reference sequence** | **Amplicon coordinates on LTP reference sequence** | **Amplicon coordinates on RDP uncultured bacteria reference sequence** |
| --- | --- | --- | --- |
| 27F+R357 | 28-342 | 19-333* | 28-342 |
| F343+R534 | 358-516 | 349-507* | 358-516 |
| F515+R806 | 534-786 | 525-777* | 534-786 |
| F784+R926 | 799-906 | 790-897* | 799-906 |
| F917+R1114 | 933-1098 | 906-1071** | 933-1098 |
| F1099+R1391 | 1115-1390 | 1088-1363** | 1115-1390 |
| F1099+R1492 | 1115-1491 | 1088-1464** | 1115-1491 |
