# Supplementary material for: Taxonomic Classification of Bacterial 16S rRNA Genes Using Short Sequencing Reads: Evaluation of Effective Study Designs
Source: PLoS One. 2013 Jan 7;8(1):e53608. doi: 10.1371/journal.pone.0053608 (PMC3538547; doi:10.1371/journal.pone.0053608)
Supplement: Table S3 — Coverage of the examined amplicons by LTP and RDP uncultured bacterial sequences. We identify each amplicon using the 16S rRNA gene hypervariable region covered by single-read sequencing configurations. We counted sequences only if they contained the entire amplicon. (DOC) [file pone.0053608.s009.doc]

**Table S3. Coverage of the examined amplicons by LTP and RDP uncultured bacterial sequences.** We identify each amplicon using the hypervariable region covered by single-read sequencing configurations. We counted sequences only if they contained the entire amplicon.

| **Amplicon** | **#LTP sequences containing amplicon** | **#LTP sequences containing amplicon and amplicons V3-V7** | **#RDP uncultured Bacteria sequences containing amplicon** | **#RDP uncultured Bacteria sequences containing amplicon and amplicons V3-V6** |
| --- | --- | --- | --- | --- |
| **V1** | 4,036 | 4,005 | 313,803 | 202,255 |
| **V3** | 8,494 | 8,394 | 1,212,547 | 854,768 |
| **V4** | 8,494 | 8,394 | 1,128,903 | 854,768 |
| **V5** | 8,494 | 8,394 | 1,066,832 | 854,768 |
| **V6** | 8,494 | 8,394 | 975,881 | 854,768 |
| **V7** | 8,394 | 8,394 | 373,575 | 292,304 |
| **V9** | 5,636 | 5,636 | 163,045 | 128,801 |
