# Supplementary material for: Taxonomic Classification of Bacterial 16S rRNA Genes Using Short Sequencing Reads: Evaluation of Effective Study Designs
Source: PLoS One. 2013 Jan 7;8(1):e53608. doi: 10.1371/journal.pone.0053608 (PMC3538547; doi:10.1371/journal.pone.0053608)
Supplement: Methods S1 — (DOC) [file pone.0053608.s017.doc]

**Taxonomic classification of bacterial 16S rRNA genes using short sequencing reads:**

**Evaluation of effective study designs**

Orna Mizrahi-Man^1^, Emily R. Davenport^1^ and Yoav Gilad^1^

^1^Department of Human Genetics, University of Chicago, Chicago IL, USA

**Supplementary Methods**

**Taxonomic annotation of the ‘unfiltered RDP’ training set**  We obtained the taxonomic annotation of the ‘**unfiltered RDP**’ training set from the RDP database records, relying on the RDP classifier hierarchy for inference of the rank of taxonomic path components. In some cases, we found that the same name is given to different levels of the hierarchy (e.g. “Actinobacteria” is both the name of a phylum and a class). To obtain the rank in these cases we relied on the parent-child relationships specified by the hierarchy and the order of names in the taxonomic path. We used the Bio::LITE::Taxonomy::NCBI module version 0.06 to extract the species name corresponding to the NCBI taxonomic identifier in the sequence record from a local copy of the NCBI taxonomy database , which we downloaded from <ftp://ftp.ncbi.nih.gov/pub/taxonomy/> on March 2, 2011. In some cases the NCBI taxonomic identifier in the sequence record no longer exists in the NCBI taxonomy databases. In cases where the identifier was merged with another taxonomic identifier (as recorded in the merged.dmp file in the database) we used the new taxonomic identifiers. However, in 17 cases the relevant taxonomic identifier was completely deleted from NCBI taxonomy (as recorded in the deleted.dmp file in the database download). We discarded these 17 cases. We also discarded the sequences matching the following conditions: no NCBI taxonomic identifier was found in the sequence record (235 sequences); the taxonomic domain in the sequence record did not match that of the NCBI taxonomic identifier (because the taxonomic identifier corresponded to the host rather than the isolate; 656 sequences); the NCBI taxonomic identifier had a rank higher than “species” (111 sequences). This resulted in taxonomic annotations for 249,687 out of 250,706 sequences.

**Length filtration of training set amplicon sequences** To ensure the high quality of the RDP sequences included in our training sets we performed length filtration of the amplicons. Our first estimate of the acceptable length range for an amplicon was based on the length distribution found in amplicons extracted from 16S rRNA genes annotated in complete genome sequences. We used the Genbank accession , associated with each RDP entry, to locate such amplicons. We complemented this estimate of amplicon length range with that obtained from the manually curated LTP bacterial sequences, setting the acceptable length range to be the maximal range covered by these two sets. Finally, to account for genera with unusual amplicon length distributions we reviewed the taxonomic distribution of the sequences whose amplicon was rejected due to the limits set by the complete genome and LTP sequences. We then adjusted the length limits to accommodate genera for which all members had amplicons that are too short or long. The length filtration resulted in loss of at most 0.16% of sequences.

**Equivalence of the RDP classifier and the mothur implementation of naiive Bayesian classifier** In this work we classified sequences using the naiive Bayesian classifier implemented in the classify.seqs function from the mothur program . According to the mothur documentation the Bayesian method implemented in classify.seqs is the same one as in the RDP classifier . We chose to use this implementation rather than the more popular RDP classifier due to the greater ease of switching training sets with it (there is no need to specify a hierarchy, rather each sequence is associated with a taxonomic path). Since the RDP classifier is much more widely used then the mothur implementation we first checked that these are indeed equivalent. To this end, we classified the full-length 8,494 bacterial LTP sequences using both the RDP classifier (v. 2.3) and the mothur implementation (v.1.20.1), training on the default RDP classifier training set (RDP training set v. 6). We then compared the results focusing on the ranks phylum, class, order, family and genus (the mothur version of this training set omits intermediate ranks, such as subclass). We found that in 8,489/8,494 cases the two classifiers fully-agreed in their classifications. In the remaining five cases the classifiers disagreed only at the genus level and the classifications obtained low confidence scores (RDP classifier: 36-49%; mothur 40-51%), indicating a choice between a number of almost equiprobable possibilities. Out of these five cases, in two both classifiers made a false prediction, in two the mothur classifier made the correct prediction, and in one the RDP classifier made the correct prediction. We also calculated the correlation between confidence scores the two classifiers obtained. Including all five ranks the correlation was 0.986, and for the individual ranks correlations were – phylum: 0.992, class: 0.987, order: 0.982, family: 0.986, genus: 0.986. In view of these results, in the remainder of the manuscript we consider these classifiers as equivalent and assume our results and recommendations apply to the RDP classifier.

**Accounting for differences between test and training taxonomies** In our leave-k-out tests the reference annotation (LTP) and all training sets except LTP differed in their underlying taxonomic hierarchy, potentially leading to over-estimation of the false prediction rate and resulting in an advantage to training sets with a taxonomic hierarchy more similar to that of the reference set. This problem was exacerbated by the usage of synonymous names for the same taxon or misspelling of taxon names.

We attempted to alleviate this situation in three ways, using comparisons of the training taxonomies with the annotation of the LTP sequence set, which served as the test set. First, we compiled a list of all synonymous or alternatively spelled taxa, creating a dictionary, which was used on the fly in the evaluation of predictions. Second, we formulated a set of rearrangement rules that would make the training taxonomy more similar to the LTP taxonomy. For example, to make the RDP database taxonomy more similar to LTP , the family “Veillonellaceae” was moved from the order “Clostridiales” to the order “Selenomonadales”. We then applied these rules to the taxonomic annotation of the training set, to obtain a rearranged taxonomy, which was then used for the classification tests. Unfortunately, these rearrangements could not account for all the differences between the taxonomies. For example, the phyla Cyanobacteria and Acidobacteria have a totally different hierarchy in the RDP database compared to LTP. In addition, we found cases where an LTP sequence was present in the training set (before removing the same-species sequences), albeit with a contradicting taxonomic annotation. Therefore, as a third measure against the over-estimation of the FPR, we compiled a list of sequences that had a conflicting annotation in at least one of the ranks. These sequences were then discounted when the FPR was evaluated for the problematic rank/s, using the union of conflicts in all training sets for the comparison of training sets and the set of conflicts found for ‘unfiltered RDP’ in later FPR calculations (no more than 4% of sequences were eliminated at any taxonomic level).

**References**

1. Cole JR, Chai B, Farris RJ, Wang Q, Kulam-Syed-Mohideen AS, et al. (2007) The ribosomal database project (RDP-II): introducing myRDP space and quality controlled public data. Nucleic Acids Res 35: D169-172.

2. Cole JR, Wang Q, Cardenas E, Fish J, Chai B, et al. (2009) The Ribosomal Database Project: improved alignments and new tools for rRNA analysis. Nucleic Acids Res 37: D141-145.

3. Sayers EW, Barrett T, Benson DA, Bolton E, Bryant SH, et al. (2011) Database resources of the National Center for Biotechnology Information. Nucleic Acids Res 39: D38-51.

4. Munoz R, Yarza P, Ludwig W, Euzéby J, Amann R, et al. (2011) Release LTPs104 of the All-Species Living Tree. Syst Appl Microbiol 34: 169-170.

5. Yarza P, Richter M, Peplies J, Euzeby J, Amann R, et al. (2008) The All-Species Living Tree project: a 16S rRNA-based phylogenetic tree of all sequenced type strains. Syst Appl Microbiol 31: 241-250.

6. Schloss PD, Westcott SL, Ryabin T, Hall JR, Hartmann M, et al. (2009) Introducing mothur: open-source, platform-independent, community-supported software for describing and comparing microbial communities. Appl Environ Microbiol 75: 7537-7541.

7. Wang Q, Garrity GM, Tiedje JM, Cole JR (2007) Naive Bayesian classifier for rapid assignment of rRNA sequences into the new bacterial taxonomy. Appl Environ Microbiol 73: 5261-5267.
